# Improved bi-allelic modification of a transcriptionally silent locus in patient-derived iPSC by Cas9 nickase

**DOI:** 10.1038/srep38198

**Published:** 2016-12-02

**Authors:** Reto Eggenschwiler, Mohsen Moslem, Mariane Serra Fráguas, Melanie Galla, Oliver Papp, Maximilian Naujock, Ines Fonfara, Ingrid Gensch, Annabell Wähner, Abbas Beh-Pajooh, Claudio Mussolino, Marcel Tauscher, Doris Steinemann, Florian Wegner, Susanne Petri, Axel Schambach, Emmanuelle Charpentier, Toni Cathomen, Tobias Cantz

**Affiliations:** 1 Research Group Translational Hepatology and Stem Cell Biology, Cluster of Excellence REBIRTH, Hannover Medical School, Hannover, 30625, Germany; 2 Department of Gastroenterology, Hepatology and Endocrinology, Hannover Medical School, Hannover, 30362, Germany; 3 Institute of Experimental Hematology, Cluster of Excellence REBIRTH, Hannover Medical School, Hannover, 30625, Germany; 4 Department of Neurology, Hannover Medical School, Hannover, 30625, Germany; 5 Max Planck Institute for Infection Biology, Department of Regulation in Infection Biology, Berlin, 10117, Germany; 6 The Laboratory for Molecular Infection Medicine Sweden (MIMS), Umeå Centre for Microbial Research (UCMR), Department of Molecular Biology, Umeå University, Umeå, 90187, Sweden; 7 Center for Chronic Immunodeficiency, Medical Center - University of Freiburg, Freiburg, 79106, Germany; 8 Institute for Cell and Gene Therapy, Medical Center - University of Freiburg, Freiburg, 79106, Germany; 9 Faculty of Medicine, University of Freiburg, Freiburg, 79106, Germany; 10 Institute of Human Genetics, Hannover Medical School, Hannover, 30625, Germany; 11 Max Planck Institute for Molecular Biomedicine, Cell and Developmental Biology, Münster, 48149, Germany

## Abstract

Homology directed repair (HDR)-based genome editing via selectable long flanking arm donors can be hampered by local transgene silencing at transcriptionally silent loci. Here, we report efficient bi-allelic modification of a silent locus in patient-derived hiPSC by using Cas9 nickase and a silencing-resistant donor construct that contains an excisable selection/counter-selection cassette. To identify the most active single guide RNA (sgRNA)/nickase combinations, we employed a lentiviral vector-based reporter assay to determine the HDR efficiencies *in cella*. Next, we used the most efficient pair of sgRNAs for targeted integration of an improved, silencing-resistant plasmid donor harboring a piggyBac-flanked puro*Δtk* cassette. Moreover, we took advantage of a dual-fluorescence selection strategy for bi-allelic targeting and achieved 100% counter-selection efficiency after bi-allelic excision of the selection/counter-selection cassette. Together, we present an improved system for efficient bi-allelic modification of transcriptionally silent loci in human pluripotent stem cells.

Precision genome engineering by HDR has been a very tedious and cumbersome endeavor in the past. Typically, thousands of cell clones had to be analyzed for detection of a single clone with a desired modification. Stimulation of HDR by the insertion of specific double-strand breaks at the genomic target locus by zinc finger nucleases (ZFN), transcription activator-like effector nucleases (TALEN), RNA-guided endonucleases (RGN) such as CRISPR/Cas9, and most recently DNA-guided endonuclease NgAgo, has considerably improved on-target efficiencies and correspondingly reduced the number of clones to be analyzed^1^,^2^. However, depending on the exact cell line and the genomic locus vast differences in targeting efficiencies have been reported^3^. Especially transcriptionally silent loci have been known as more difficult targets for HDR- and non-homologous end joining (NHEJ)-based genome modification^3^,^4^,^5^. For HDR-based precision genome engineering in cell culture mainly two different kinds of donor templates are commonly being used these days: double-stranded selectable long flanking arm donors with typically >400 bp left and right flanking arms or short single-stranded oligodeoxynucleotides (ssODNs). Less frequently, recombinant adeno-associated vectors (rAAV) have been employed as HDR donors, which are similar in structure to long flanking arm donors but are particularly suitable for *in vivo* HDR approaches^6^. Of the two more common strategies, ssODNs are considerably smaller than long flanking arm donors, typically 50–200 bp of total length, and they are widely being used for insertion or deletion of small fragments or for correction of single base pair mutations^7^. However, ssODNs are quite restricted in size and capacity rendering them incapable of carrying large selection cassettes and thus limiting selection-based approaches to genetic modification which result in inherent growth advantage under certain growth conditions, such as HGPRT mutant cell lines in HAT medium^8^. Therefore, using ssODNs for HDR-based genome engineering still requires tedious analysis of hundreds of clones, in order to identify few bi-allelic targeted ones^9^. Moreover, technical limitations restrict the size of insertions or deletions which can be introduced using ssODNs, whereas plasmid donors allow for more flexibility and can carry larger inserts such as selection cassettes. Using recognition sites for transposases with small footprints such as piggyBac or sleeping beauty, selection/counter-selection cassettes such as the fusion of the puromycin-resistance gene with the herpes simplex virus thymidine kinase (puro*Δtk*) can subsequently be seamlessly excised^10^,^11^. However, for an effective approach the selection/counter-selection genes need to be expressed at sufficient level and most of all, expression of the counter-selection gene should not depend on the addition of a primary selector, such as puromycin in the case of the puro*Δtk* fusion construct. Therefore, a strong and silencing-resistant promoter is desirable for expression of such a cassette, especially at silencing-prone genomic loci.

Here, we targeted the transcriptionally silent *SERPINA1* locus in human iPSC derived from a patient with severe ZZ α1-antitrypsin (AAT) deficiency using Cas9 nickase and single guide RNAs (sgRNAs) with a long flanking arms donor. Cas9 nickase was previously shown to be highly efficient for targeted knock-in of HDR donors, while off-target mutations were found to be considerably lower than when using Cas9 nuclease^12^. Moreover, “double-nicking” with two sgRNAs on opposite strands of DNA was shown to be a highly efficient method for targeted knock-ins, likely due to the fact that part of the DNA re-section process is already performed by the nature of the double strand separation introduced with a double-nick^13^. However, the distance between the two nicks as well as the specific molecular nature of the DNA at the location where the double-nick seem both to have a vast influence on targeting efficiencies which can be achieved with a HDR donor^14^. Therefore, a simple evaluation of the double-strand cleavage efficiency of different sgRNAs via *in vitro* assays such as SURVEYOR or T7 can only partially predict the actual efficiency which can be achieved by double-nicking. For a more accurate estimation, we here suggest the evaluation of sgRNA combinations by an *in cella* assay where the target sequence is introduced into an inactivated fluorescence reporter into the genome which can then be re-activated by an appropriate donor upon stimulation of HDR by Cas9 nickase. To this end, we designed four sgRNAs targeting *SERPINA1* and tested them *in cella*. Using two sgRNAs with high on-target efficiency together with a previously published excisable selection/counter-selection plasmid donor^10^,^11^, we achieved bi-allelic targeting in up to 40% of all clones but we also observed vast transgene silencing. We then directly compared transgene expression driven by the mouse PGK promoter used in this donor with the one of the CAG (CMV i/e enhancer-chicken β-actin promoter-rabbit globin intron) promoter in human pluripotent stem cells and we found large differences in their stabilities. With the more silencing-resistant CAG promoter and two different fluorescence markers we identified bi-allelic targeted clones in which we achieved 100% fialuridine (FIAU)-based counter-selection efficiency in a cell line where the commonly used PGK promoter had failed for that purpose.

Together, our work presents an improved method for direct bi-allelic targeting followed by one-step bi-allelic excision of selection/counter-selection cassette at a transcriptionally silent locus in human pluripotent stem cells. Moreover, our report also highlights the importance of functional evaluation of sgRNAs for CRISPR/Cas9-based HDR stimulation *in cella*.

## Results

### A lentiviral-based system for the *in cella* evaluation of HDR induction efficiencies of different sgRNA combinations with Cas9 nickase

**Figure 1:**
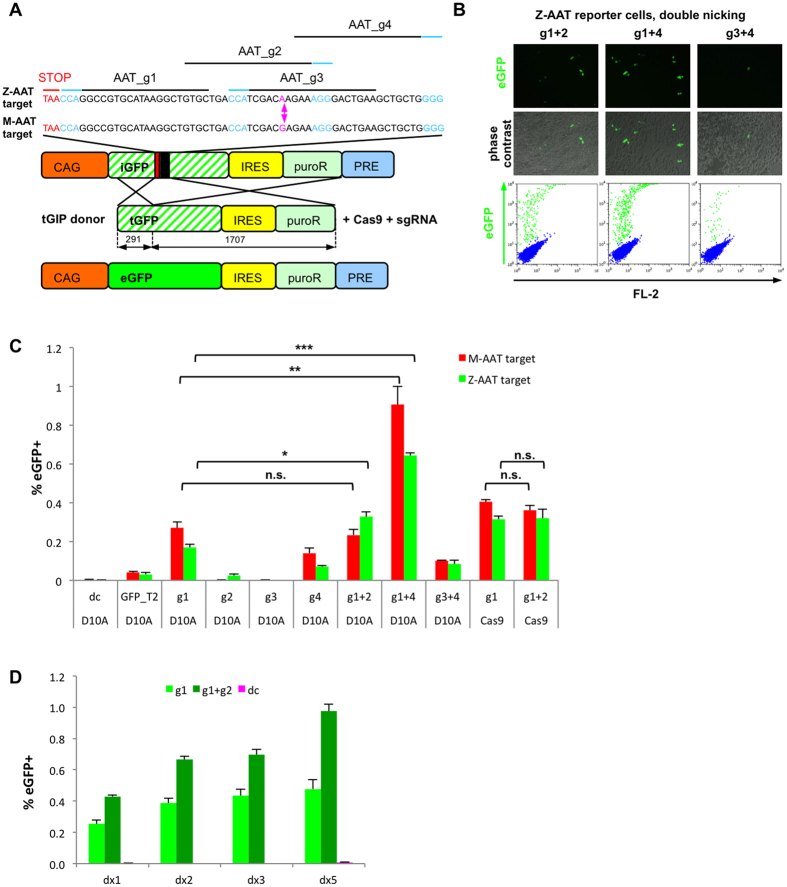
Assessment of HDR-stimulation efficiency of sgRNAs by a lentiviral vector-based reporter assay. (**A**) An eGFP cassette was interrupted by a STOP codon (iGFP) and by the targeting sequence for either Z-AAT or M-AAT, which differ by the G > A polymorphism (pink). PAM’s are denoted in blue. AAT_g2 and AAT_g3 sgRNAs both span the single nucleotide difference. Reporter cells generated by transduction at low MOI and puromycin selection can be transfected by promoterless truncated GFP-IRES-puroR (tGIP) donor + Cas9 + sgRNAs for analysis of HDR efficiency in cellam. (**B**) Representative fluorescence microscopy pictures (upper panel) and FACS analysis (lower panel) of HEK Z-AAT-target reporter cells transfected with tGiP, Cas9_D10A nickase and different sgRNA combinations resulting in generation of 5′ overhangs. (**C**) FACS analysis of HEK reporter cells transduced with either M-AAT or Z-AAT target and transfected with Cas9_D10A nickase (D10A) or Cas9 nuclease (Cas9) and different sgRNAs or sgRNA combinations. Cells transfected with ubiquitous GFP_T2 sgRNA and cells transfected with donor and Cas9_D10A only (dc) served as positive and negative controls, respectively. (**D**) Increasing frequency of homology directed repair for single and for double nicking by increase of tGIP donor : Cas9 : sgRNA ratio from 1:1:1 (dx1) to 5:1:1 (dx5). Data are represented as mean ± SD and statistical analysis was performed by two-tailed student’s t-test where *p < 0.05, **p < 0.01, ***p < 0.001, n.s. = not significant, n = 3 biological replicates.

Stimulation of HDR at a genomic target locus requires efficient cleavage in combination with a suitable homologous DNA donor^15^. RGNs use small RNAs as guide sequence for specific cleavage of genomic DNA at any locus which is next to a protospacer adjacent motif (PAM). Commonly used RGNs such as SpCas9 harbor two active domains for complete cleavage of the double strand: the RuvC domain cleaves the DNA strand complementary to the gRNA whereas the HNH domain cleaves the opposite strand^16^. Inactivation of either the RuvC domain by D10A mutation or of the HNH domain by H840A or N863A mutation results in a nickase which can cleave only one of the strands. When using 2 opposite-strand sgRNAs with Cas9 nickase, it is possible to open the genomic DNA with an overhang, which can then strongly stimulate HDR at the target locus. In order to test the magnitude of this stimulation by different sgRNA combinations, we cloned a lentiviral reporter vector based on a previously described construct^17^ and we inserted a 59 bp stretch of the genomic target locus inside an eGFP sequence interrupted by a stop codon (iGFP), leading to a frameshift (Fig. 1A). Here, we chose to target Exon V of the human *SERPINA1* gene encoding for α1-antitrypsin (AAT). A well described G > A point mutation on base number 31 of this Exon leads to a missense amino acid replacement (E342K) resulting in expression of Z-AAT, which is the underlying cause for clinical severe AAT deficiency^18^. In order to test whether it was possible to use the G > A point mutation for allele-specific targeting of the Z-AAT allele, we cloned either the 59 bp target sequence of Z-AAT or the target sequence of the normal *SERPINA1* allele (M-AAT) into the reporter vector. We used both vectors separately to transduce HEK 293 T cells at low MOI and selected the cells using puromycin and we analyzed the vector copy numbers of the resulting bulk populations (Supplementary Fig. S1C). The iGFP in these cells can be corrected with a promoter-less, truncated eGFP-IRES-puro donor (tGIP) upon stimulation of HDR by sequence-specific cleavage. Using these reporter cell populations we first compared different nickases D10A, H840A and N863A for their ability to stimulate HDR in our reporter system. We found that with both, AAT_g1 sgRNA and a previously published eGFP-specific sgRNA (GFP_T2, refs 12 and 19), RuvC-deficient D10A nickase was superior to the two HNH-deficient nickases (Supplementary Fig. S1D). Next, we evaluated stimulation of HDR upon transfection of Cas9 nuclease or Cas9_D10A nickase together with different sgRNAs (Fig. 1B,C). HDR efficiencies were slightly higher in Cas9 nuclease treated cells compared to nickase treated cells when using the most efficient AAT_g1 sgRNA. However, there was no further increase in efficiency when using Cas9 nuclease in concert with two sgRNAs AAT_g1 and AAT_g2, whereas we observed a significant increase in eGFP+ cells when using these two sgRNAs with Cas9_D10A nickase on the Z-AAT target transduced cells and we observed an even stronger increase when using AAT_g1 together with AAT_g4 sgRNA, compared to cells transfected only with AAT_g1 sgRNA. Importantly, the combination of the latter two sgRNAs lead to an increase of efficiency which is much higher than simple addition of single efficiencies of AAT_g1 and AAT_g4, suggesting sequence-specific synergistic effects, which could clearly be detected in the context of this *in cella* reporter system. AAT_g2 and AAT_g3 sgRNAs notably fully match the Z-AAT target, whereas they have a mismatch for the M-AAT target at the indicated site (Fig. 1A). To our surprise, we found AAT_g3 sgRNA did not seem to stimulate HDR at the target locus at all and we neither found a significant difference in appearance of eGFP+ cells compared to donor control nor could we improve the targeting of AAT_g4 sgRNA by double nicking with AAT_g3 sgRNA (Fig. 1C). Nevertheless, we observed allele-specific targeting when using AAT_g2 sgRNA at a low but consistent efficiency (Fig. 1C and Supplementary Fig. S1A). We further confirmed the allele-specific cleavage in an *in vitro* digestion assay using purified Cas9 protein together with tracrRNA and AAT_g2_crRNA (Supplementary Fig. S1B). Moreover, we analyzed the dose-dependency of donor addition and we observed a strong increase in targeting efficiency when adding increasing amounts of tGIP donor per Cas9_D10A and sgRNA in both, single and double nicking assays (Fig. 1D). On a side note, we observed a slightly higher targeting at the M-AAT target compared to the Z-AAT target for all sgRNAs except for the allele-specific AAT_g2 sgRNA, which most likely resulted from the higher VCN in M-AAT cells (Supplementary Fig. S1C). Finally, we analyzed whether the formerly tested less efficient nickases H840A and N863A could as well have higher HDR-stimulation efficiency when using two opposite strand sgRNAs, but we found no increase for double nicking with AAT_g1 and AAT_g2 sgRNAs, which creates a 3′ DNA overhang. Double nicking with H840A or N863A and AAT_g1 and GFP_T2 sgRNAs however, resulted in a 5′ overhang, which additionally stimulated HDR (Supplementary Fig. S1E). We additionally tested the usability of this reporter system for two additional disease-related genomic loci: the V30M mutation of the TTR gene which is well documented for familial amyloid polyneuropathy (FAP) and the D90A and R115G mutations in the SOD1 gene which are associated with amyotrophic lateral sclerosis (ALS). To this end, we replaced the Z-AAT target sequence in the lentiviral reporter vector with the corresponding genomic sequences of TTR or SOD1, respectively and we designed sgRNAs matching alongside the new targets (Supplementary Table S4). Amongst four tested TTR sgRNAs we found that nicking with TTR_g1 sgRNA resulted in increased HDR stimulation compared to GFP_T2 sgRNA and that all double-nicking combinations resulting in a 5′ overhang showed much higher efficiencies than separate sgRNAs (Supplementary Fig. SF). For the SOD1 target we tested five sgRNAs and nicking with SOD1_g4 sgRNA stimulated HDR more than compared to standard GFP_T2 sgRNA. Notably, double nicking with SOD1_g4 and SOD1_g5 sgRNAs resulted in a very short 14 bp 5′ overhang, which seemed to be not sufficient for additional stimulation of HDR. Combination of SOD1_g3 and SOD1_g5 sgRNAs, however, generated a 57 bp 5′ overhang and we observed a strong increase of HDR. Importantly, HDR stimulation of SOD1_g5 sgRNA alone was indistinguishable from donor control transfected cells without sgRNA, which again highlights the importance of *in cella* assays for double nicking approaches (Supplementary Fig. S1G).

### Efficient bi-allelic targeting of a transcriptionally silent genomic target locus and rapid silencing of the selection/counter-selection cassette

**Figure 2:**
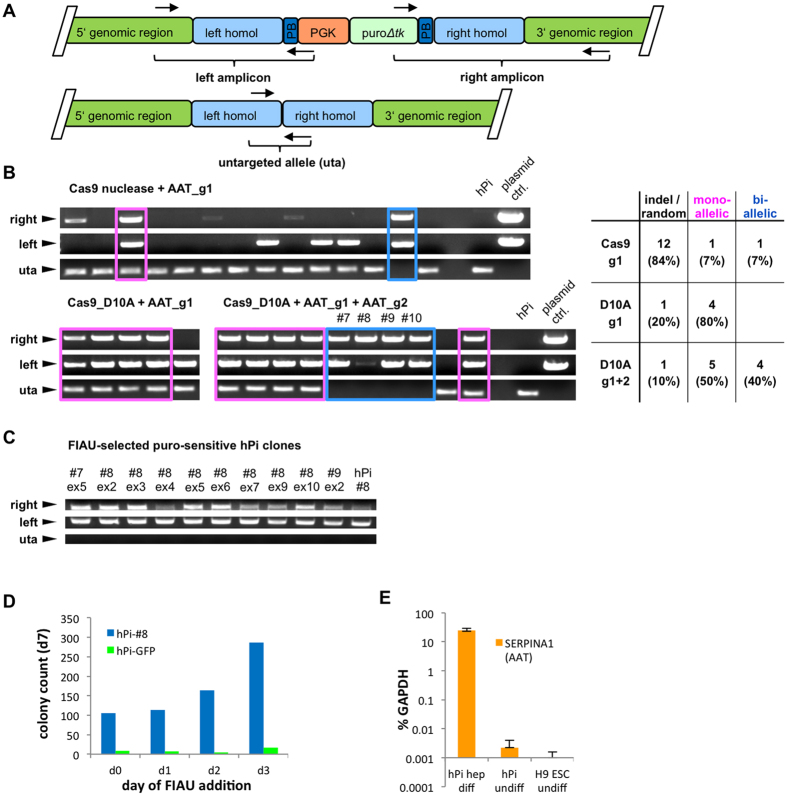
Efficient bi-allelic targeting in human iPSC and problematic FIAU-based counter-selection. (**A**) Schematic of correctly integrated AAT-PB-PGK-puroΔtk donor in the human *SERPINA1* locus and of untargeted allele. (**B**) PCR analysis of puromycin selected human iPSC clones from a patient with severe ZZ-A1AT deficiency (hPi) transfected with and Cas9 nuclease + AAT_g1 or Cas9_D10A + AAT_g1 or Cas9_D10A + AAT_g1 + AAT_g2 gRNAs. Primers for detection of integrated flanking arms are matching the genomic regions up- and downstream of the flanking arms, respectively, and inside the selection cassette. Primers for detection of untargeted allele (uta) generate a short fragment in the genomic region corresponding to the left and right flanking arms. Mono-allelic targeted clones show all three bands: left, right and uta (framed in pink), whereas bi-allelic targeted clones show absence of the uta band (framed in blue). Clones with random integration or with distorted integration at the target locus show either no left band, no right band or none of both. Plasmids containing cloned sequences of integrated left or right flanking arms including the primer matching sites and gDNAs from parental hPi cells served as controls. (**C**) Bi-allelic targeted clones hPi-#7, hPi-#8 and hPi-#9 were transfected with hyperactive piggyBac transposase and selected using FIAU. FIAU-resistant, puro-sensitive clones were analyzed by the same PCRs as in (**B**). (**D**) Colony formation of bi-allelic targeted clone hPi-#8 at different days of FIAU addition post puromycin withdrawal. At day zero cells were directly transferred from puromycin to FIAU medium. A 1% spike of a HSV thymidine kinase-negative hPi-derived cell line with stable eGFP expression (hPi-GFP) served as control. (**E**) QRT-PCR analysis of AAT expression levels in undifferentiated hPi iPSC and H9 ESC compared to d17 hepatic differentiated hPi, relative to GAPDH. Data in (E) are represented as mean ± SD with n = 3 biological replicates.

After successful evaluation of AAT sgRNAs we attempted correction of the human Z-AAT gene in a previously published patient-specific iPSC line (hPi) generated from an individual with severe ZZ-AAT deficiency^20^. This cell line is homozygous for the E342K missense point mutation and has been used for functional hepatic disease modeling studies. We first used the most efficient AAT sgRNA, AAT_g1, together with Cas9 nuclease and a previously published long flanking arms donor construct harboring a piggyBac site-flanked puro*Δtk* selection/counter-selection cassette (AAT-PB-PGK-puro*Δtk*, ref. 10). Transfected and puromycin selected hPi iPSC clones were analyzed for integration of the left and right flanking arms by PCR as depicted in Fig. 2A. We found that one out of 14 clones was correctly targeted on one allele and one clone was targeted on both alleles while six clones were targeted by random integration and further six clones showed only either left or right flanking arm amplicon, suggesting that potential Cas9 nuclease-induced deletions at the other respective flanking arm may have impaired the primer binding sites (Fig. 2B). Next, we used Cas9_D10A nickase with AAT_g1 sgRNA and isolated five clones, four of which were targeted on one allele. However, as the diseased allele is homozygous, bi-allelic targeting for complete correction is needed. To increase targeting frequency we next employed two sgRNAs. We had previously shown that combination of AAT_g1 + AAT_g4 was most efficient (Fig. 1C). However, since AAT_g4 has considerably higher off-target score than allele-specific AAT_g2 (Supplementary Table S2), we decided to continue with the slightly less efficient but more safe combination of AAT_g1 + AAT_g2 sgRNAs. Out of 10 puromycin-resistant clones generated by double nicking, five were targeted on one allele and four were targeted on both alleles, displaying a 40% bi-allelic targeting efficiency (Fig. 2B). We transfected three of the bi-allelic targeted cell lines with an expression plasmid for piggyBac transposase for excision of the selection/counter-selection cassette and selected them against HSV-thymidine kinase with FIAU from day 4 post transfection, according to a previously published protocol^10^. After this selection step, we picked a total of 30 FIAU-resistant colonies (10 of each parental line) and analyzed if the selection/counter selection cassette was successfully removed. We first probed all 30 clones for remaining puromycin resistance and identified 20 clones, which were puromycin-resistant and thus still harbored the selection cassette. However, much to our surprise none of the 10 remaining FIAU-resistant and puro-sensitive clones showed disappearance of the flanking arms bands or re-appearance untargeted allele (Fig. 2C). In order to investigate whether the time-point of FIAU addition after puromycin withdrawal could make a difference for the selection procedure, we analyzed colony-formation efficiency of one bi-allelic targeted cell line under FIAU-addition at different days after puromycin withdrawal. In this assay, we observed that many colonies of the bi-allelic targeted cell line were surviving even when we transferred the cells directly from puromycin-containing to FIAU-containing medium and that this effect was increasing with later time-points of FIAU-addition (Fig. 2D). In order to test the overall integrity of the puro*Δtk* cassette, we added puromycin and FIAU together and we saw that all three bi-allelic targeted cell lines readily died off within 5 days of combined selector addition. Therefore, the FIAU resistance observed in non-excised bi-allelic targeted cell lines must have been acquired through rapid silencing of the mouse PGK promoter which was used in the donor construct. Moreover, qRT-PCR analysis of AAT expression showed that the *SERPINA1* locus is transcriptionally silent in human pluripotent stem cells, which could be associated with observed silencing effects (Fig. 2E).

### A more robust and traceable selection/counter-selection cassette with 100% counter-selection efficiency

**Figure 3:**
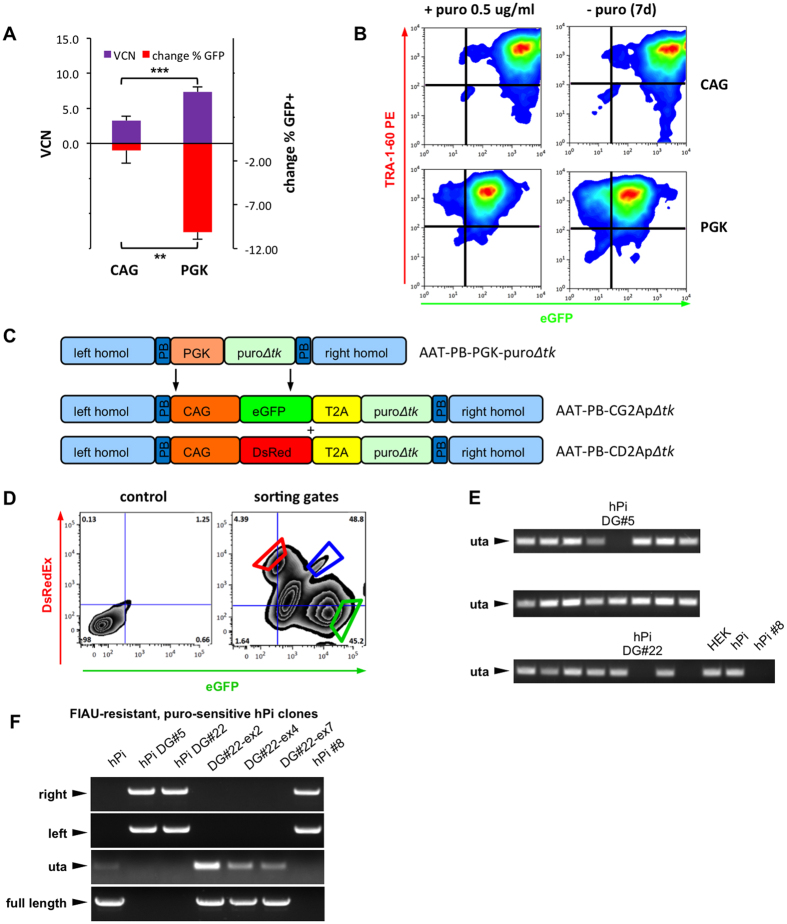
A stronger and more silencing-resistant construct for efficient selection of bi-allelic excised iPSC clones. (**A**) Analysis of transgene silencing in the TRA-1–60^+^ population of CAG-eGFP-IRES-puro or PGK-eGFP-IRES-puro transduced and puromycin-selected hPi cells, 7 days after puromycin withdrawal. Difference between identical bulk populations cultivated with and without puromycin was calculated, giving the % of transgene silencing. Average lentiviral vector copy numbers (VCN) were determined by qRT-PCR. (**B**) Representative FACS plots from CAG- or PGK-transduced hPi cells, grown for 7 days with or without puromycin. FACS-controls are given in Figure S2A. (**C**) Schematic of the new selection/ counter-selection cassette: the PGK promoter in the AAT-PB-PGK-puro*Δtk* plasmid was replaced by CAG and an eGFP or DsRedEx cassette was added over a T2A joint before the puro*Δtk* for better monitoring of transgene expression, resulting in AAT-PB-CG2Ap*Δtk* or AAT-PB-CG2Ap*Δtk*, respectively. (**D**) FACS sort for DsRed^+^ single positive (framed in red), eGFP^+^ single positive (framed in green) and DsRed^+^, eGFP^+^ double positive (framed in blue) hPi cells after transfection of AAT-PB-CG2Ap*Δtk* + AAT-PB-CD2Ap*Δtk* + Cas9_D10A + AAT_g1 + AAT_g2 and puromycin selection. (**E**) PCR analysis for the untargeted *SERPINA1*-allele in 23 established clonal cell lines generated from double-positive sorted cells. Genomic DNA from HEK293-T and hPi parental cell lines served as positive controls, whereas bi-allelic targeted hPi #8 was used as negative control. (**F**) PCR analysis of piggyBac excised, FIAU-resistant and puromycin-sensitive clones generated from bi-allelic targeted clones for left and right flanking arm bands and untargeted or excised allele (uta). Presence or absence of full length 2.5 kb genomic fragment spanning over the genomic location of both flanking arms was analyzed using forward primer from left band PCR and reverse primer from right band PCR. hPi and hPi #8 served as controls. Data are represented as mean ± SD and statistical analysis was performed by two-tailed student’s t-test where **p < 0.01, ***p < 0.001, n = 4 biological replicates (= individual transductions).

In previous studies, we had compared the EF-1α short promoter (EFS), the full length EF-1α promoter and the CAG promoter for their resistance to transgene silencing in mouse iPSC^20^. We found that CAG was the most stable of these promoters and expression persisted even throughout differentiation *in vitro* and *in vivo*, and that expression was also stable in human iPSC even at low lentiviral vector copy numbers (VCN). Therefore, we decided to directly compare the PGK promoter from AAT-PB-PGK-puro*Δtk* with the CAG promoter for stability of transgene expression in human pluripotent stem cells. To this end, we transduced hPi cells with lentiviral vectors encoding for an eGFP-IRES-puro cassette driven either by PGK or CAG promoter and selected them with puromycin. After 6 days, all four individually transduced bulk populations were split onto two wells each, while one well was kept on puromycin and one well was cultivated in the absence of puromycin for 7 days. FACS analysis of eGFP+ cells in the TRA-1–60 expressing population revealed substantial silencing of the PGK promoter whereas we observed no notable reduction in eGFP+ cells in the CAG transduced bulk populations (Fig. 3A,B and Supplementary Fig. S3A). Moreover, the CAG transduced cells showed higher fluorescence intensity than cells transduced with the PGK promoter, while a qPCR assay for VCN determination revealed less than half as many vector integrations in the lenti-CAG compared to lenti-PGK transduced bulk population (Fig. 3A,B). With these results at our hand we felt confident that we had found a better, more silencing resistant promoter and we replaced the PGK promoter by the CAG promoter in the selection/counter-selection cassette in the long flanking arms donor. Moreover, we added either an eGFP or a DsRedEx fluorescence marker via a T2A site upstream of the puro*Δtk* cassette for easier monitoring of transgene expression (Fig. 3C). We used the new AAT-PB-CG2Ap*Δtk* construct together with Cas9_D10A nickase and AAT_g1 + AAT_g2 sgRNAs for targeting of the human AAT locus in hPi cells but to our surprise, in three separate attempts and a total of 33 individual puromycin-resistant cell lines, we were not able to achieve bi-allelic targeting (Supplementary Fig. S3B). In order to help enforcing bi-allelic targeting with the new donor construct, we reasoned that a dual-fluorescence approach using a combination of both, eGFP- and DsRedEx-containing donors, might present a valid strategy. Presence of two fluorescence markers within the same clone should indicate that the corresponding cell line has integrated one fluorescence marker on each allele. To this end, hPi cells were transfected with both donors and Cas9_D10A nickase and AAT_g1 and AAT_g2 sgRNAs and selected using puromycin followed by FACS sorting for the eGFP+, DsRed+ double positive fraction (Fig. 3D). Sorted double positive cells were plated as single cells and individual colonies were analyzed by PCR for remnants of untargeted *SERPINA1* alleles. Two out of 23 analyzed clones showed complete absence of the PCR product and, thus, were likely targeted on both alleles (Fig. 3E). Amplification and sequencing of the left and right flanking arms confirmed flawless integration in both clones (Fig. 3F). We subjected the two bi-allelic targeted iPSC lines hPi-DG#5 and hPi-DG#22 to hepatic *in vitro* differentiation and we found that AAT protein was absent at the end of differentiation, confirming a full “knockout” phenotype (Supplementary Fig. S6A). Moreover, we predicted gRNA off-targets by the Zhang lab algorithm (http://crispr.mit.edu/)^21^, and we amplified and sequenced the top 5 off-target sites for AAT_g1 and the top 2–6 off-targets sites for AAT_g2 sgRNA (using the standard human genome as reference sequence, the first predicted off-target for AAT_g2 is *SERPINA1* itself, since there is one mutation in the input Z-AAT target sequence; Supplementary Table S2) in both clones and we found no change in the sequence compared to the parental hPi cell line. We also sorted for DsRed+ as well as eGFP+ single positive fractions (Fig. 3D) and analyzed 12 clones each. As expected, we did not find bi-allelic targeting in these clones (Supplementary Fig. S5B). However, most of these clones as well as most of the remaining double positive clones were correctly targeted on the respective allele (Supplementary Fig. S5C). We additionally evaluated fluorescence images of bi-allelic targeted clones hPi-DG#5 and hPi-DG#22 and we found that the cells were positive for both, eGFP and DsRedEx. As the DsRedEx signal was very weak for visual detection, we compared bi-allelic targeted cells to mono-allelic targeted cell line hPi-G#1 which was isolated from the eGFP+ single positive fraction. In spite of the high background fluorescence in the DsRedEx channel, bi-allelic targeted cell lines were still clearly distinguishable from the eGFP single positive cell line (Supplementary Fig. S5D). In order to further substantiate targeting of DsRedEx and eGFP donor plasmids in the bi-allelic targeted cell lines, we established specific PCRs for both, eGFP and DsRedEx, and we saw presence of both bands in bi-allelic targeted clones, whereas in hPi-G#1 only the eGFP band appeared (Supplementary Fig. S5E). Moreover, we confirmed bi-allelic targeting by two Southern blots. In the first blot we digested the genomic DNA using EcoRI and hybridized it with probe #2 which binds downstream of the right flanking arm. The selection/counter-selection cassette introduces an additional EcoRI site into the targeted allele, which results in detection of a lower, ~5.5 kb band in targeted alleles, whereas untargeted alleles show a higher, ~9.6 kb band (Supplementary Fig. S5F, S5G). In the second blot, we used BspHI for gDNA digest and hybridized with probe #1 (binding upstream of left flanking arm), which results in a ~8.8 kb band for untargeted and ~13.8 kb band for targeted alleles (Supplementary Fig. S5F, S5H). Both Southern blots confirmed correct bi-allelic targeting of hPi-DG#5 and hPi-DG#22. Of note, we also analyzed mono-allelic hPi-G#1 on the same blots, but unfortunately, DNA quality was not good enough to give a clear signal (Supplementary Fig. S5J,K). Finally, we transfected bi-allelic targeted cell line hPi-DG#22 with piggyBac transposase for excision of the selection cassette and selected the cells with FIAU. We established three FIAU-resistant, puro-sensitive cell lines, DG22-ex2, DG22-ex4 and DG22-ex7, which we examined by PCR. All three clones were excised on both alleles and sequencing revealed correction of Z-AAT point mutation as well as the silent mutation caused by the piggyBac footprint, indicating 100% counter-selection efficiency (Fig. 3F, Supplementary Fig. S5A). However, analysis of excised cell lines by Southern blot revealed a mono-allelic aberration at the *SERPINA1* locus in clone DG22-ex4, which was clearly visible in both blots (Supplementary Fig. S5G,H). We did not observe this aberration in parental hPi-DG#22 clone nor in its siblings DG22-ex2 and DG22-ex7, which is why we suspect that this must have happened during the piggyBac excision step rather than in the Cas9-based targeting step. Moreover, we detected neither eGFP nor DsRedEx in a PCR analysis of this clone (Supplementary Fig. S5E). Together with the fact that Southern blot probe #1 upstream and probe #2 downstream of the modified sequence both bind to the aberrant allele fragment this suggests that the selection/counter-selection cassettes have been excised in DG22-ex4 but that there were some unknown changes in close proximity of the gene-edited locus. Nevertheless, clones DG22-ex2 and DG22-ex7 both display flawlessly corrected *SERPINA1* alleles (Supplementary Fig. S5E,G,H). We also analyzed genomic integrity of gene corrected cells by Array-CGH and we found that overall, the genomes were intact (Supplementary Table S7, Supplementary Fig. S8A). However, there were a total of four differences detected between parental hPi and gene corrected cells. Three of these most likely appeared due to sensitive threshold settings and were not considered a concern after a close-up visual evaluation (Supplementary Fig. S8C–E). However, there was a partial loss of an ~30 kb fragment on chromosome 1 in gene corrected cell lines. We suspect that this may have happened during cell culture of the parental targeted clone, as all three gene corrected cell lines show the same aberration (Supplementary Fig. S8B). Favorably, no known coding regions were affected, and thus, this small aberration would most likely not have functional consequences for the gene corrected cells. Finally, we also assessed expression of pluripotency markers OCT4, SOX2 and NANOG in targeted and in gene corrected cell lines and we found no significant changes compared to parental hPi cell line (Supplementary Fig. S6B). Also, expression of TRA-1–60, which is often used as a surface marker for pluripotent cells, was not affected in gene corrected cell lines (Supplementary Fig. S6C).

### Functional correction of disease phenotype in gene edited iPSC clones

**Figure 4:**
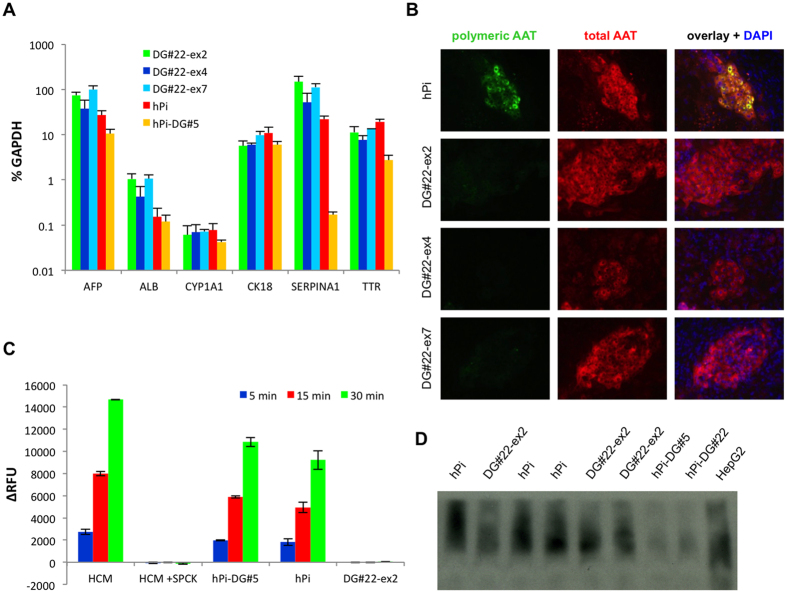
Functional correction of the Z-AAT phenotype in *in vitro* hepatic differentiated gene-corrected iPSC. (**A**) Taqman-based qRT-PCR analysis for hepatic markers of day 17 hepatic differentiated DG#22-ex2, DG#22-ex4, DG#22-ex7, hPi and hPi-DG#5 cell lines, relative to GAPDH. (**B**) Co-staining of hepatic differentiated corrected (DG#22-ex2, DG#22-ex4, DG#22-ex7) and uncorrected parental hPi cell lines for polymeric AAT using a polymer-specific antibody (2C1) and for total AAT. (**C**) Neutrophil elastase inhibition assay of 24 h supernatants of day 17 hepatic differentiated gene corrected DG#22-ex2, parental hPi and non-excised bi-allelic targeted hPi-DG#5 cell lines. Hepatocyte culture medium (HCM) and HCM with 60 uM (final) SPCK inhibitor served as negative and positive controls, respectively. Progression of fluorescence was measured after 5, 15 and 30 min with n = 3. (**D**) Native Western blot for detection of high molecular AAT protein in hepatic differentiated corrected cell line DG22-ex2 compared to Z-AAT expressing hPi parental cell line. HepG2 hepatocarcinoma cells and hepatic differentiated AAT-knockout cell lines hPi-DG#5 and hPi-DG#22 served as positive and negative controls, respectively. Data are represented as mean ± SD with n = 3 biological replicates.

In order to analyze functional correction of the Z-AAT related disease phenotype, we differentiated the gene corrected cell lines using a hepatic differentiation protocol. We first analyzed a set of hepatic markers by qRT-PCR and we found comparable expression levels in all cell lines (Fig. 4A). We also included bi-allelic targeted, non-excised hPi-DG#5 cell line in the assay as a control and we detected very low levels of AAT (*SERPINA1*), which was most likely due to the fact that the insertion of the selection cassette is in Exon V, whereas the used TaqMan^®^ assay amplifies a region between Exons II and III, where it could detect low levels of transcript remnants. Furthermore, we stained hepatic derivatives of the corrected cell lines for polymeric AAT using the polymer-specific 2C1 antibody, which has been established as gold standard for detection of AAT polymers by David Lomas lab^22^ and we co-stained for total AAT with a separate antibody. Whereas parental hPi cell line showed presence of polymeric AAT, all three corrected cell lines did not (Fig. 4B). For functional characterization of gene corrected cells we also subjected supernatants harvested from differentiated DG22-ex2 cell line to neutrophil elastase inhibitory assay and we found that secreted AAT was fully inhibiting the enzyme, whereas supernatants from parental hPi and from non-excised hPi-DG#5 did not (Fig. 4C). Z-AAT is technically able to functionally inhibit neutrophil elastase, however, it cannot be secreted very well, which may help to explain the big difference between corrected and uncorrected cells. Finally, we submitted cell lysates from differentiated corrected and uncorrected cells to native Western blot and we found that AAT from corrected DG22-ex2 cells runs at lower height, clustering in the same range as AAT isolated from HepG2 hepatocarcinoma cells, whereas AAT from uncorrected parental hPi cells was found at higher molecular weight, indicating multimerization typical for Z-AAT (Fig. 4D). The same samples were loaded in an SDS gel as well for assessment of loading control (Supplementary Figure S6A). Together, these results confirm functional correction of *in vitro* disease phenotype in bi-allelic corrected cell lines.

## Discussion

Induction of DNA double strand breaks or nicks by RGNs at genomic target loci has paved the path for efficient and highly versatile precision genome engineering. Especially HDR-based genome editing with homology donors can be greatly stimulated by double-nicking strategies using two opposite strand sgRNAs for generation of 5′ overhangs^14^. Moreover, Cas9 nickase was shown to cause significantly less off-target mutations than Cas9 nuclease^12^, as DNA nicks can be re-sealed by repair enzymes such as DNA Ligase I, whereas double-strand breaks are often dependent on more error-prone pathways, such as NHEJ^23^. In our work, we did not detect mutations at any of the top-five predicted off-target sites of the sgRNAs used for genome editing, which supports this view. However, different sgRNAs can have different individual efficiencies, and moreover, their combined efficiency for double-nicking approaches was shown to be dependent on the number of base pairs of offset between them and the exact molecular configuration in the immediate neighborhood^14^. Therefore, it is essential to evaluate HDR-stimulation efficiencies of sgRNA combinations for double-nicking approaches not only in *in vitro* assays such as T7 endonuclease assay but also in a more representative reporter system *in cella*. Perhaps *in vitro* assays may be less time consuming than the method we chose to use here, but when it comes to approaches involving double-nicking of DNA, a simple assessment of crude cleavage efficiency may be not representative for the most efficient sgRNA combinations for the induction of HDR. Here, we found sequence-specific synergistic effects and a strong increase in HDR efficiency with certain sgRNA combinations, which would go undetected in raw *in vitro* assays. To this end, we have designed a reporter vector harboring the beginning of Exon V of the human *SERPINA1* locus and we evaluated the individual and combined efficiencies of sgRNAs for induction of HDR. Several mutations at the *SERPINA1* locus have been described to be the causative for AAT deficiency and particularly the E342K point mutation at the beginning of Exon V is related with a severe clinical course. Patients often present a progressive lung disease, such as chronic obstructive pulmonary disease (COPD) and a fraction of them present variable degrees of chronic liver damage, ranging from mild fibrosis to cirrhosis or even hepatocellular carcinoma^18^. E342K-AAT is prone to self-agglutination and cannot be properly secreted from hepatocytes in the liver, where accumulation inside the endoplasmic reticulum can cause liver disease, whereas lung disease is a consequence of severely reduced AAT serum-concentration. Therefore, this mutation results in both, a ‘loss-of-function’ as well as a ‘gain-of-function’ phenotype. Compared to E342K homozygous individuals, heterozygous carriers have a strongly reduced chance for both, chronic lung and liver disease, however they are still significantly more prone to develop clinical complications than unaffected individuals^24^, which is why a bi-allelic correction of this mutation is required. Therefore, it was essential to identify sgRNA combinations which are most efficient for stimulation of HDR. In our analysis, we observed that combinations of sgRNA, which generate a 5′ overhang when used for double-nicking with D10A, H840A or N863A nickases, result in overall HDR efficiencies which are higher than the sum of individual sgRNA efficiencies, suggesting synergistic effects. This is also consistent with previous findings^14^. To our surprise one of the tested sgRNAs did not stimulate HDR at all. At this point we can only speculate about the exact reasons behind this, but it is possible that a sequence-specific secondary or tertiary structure sterically inhibited AAT_g3 sgRNA from binding to the target locus. Importantly, we used two sgRNAs with predicted low off-target activity for genome engineering of the *SERPINA1* locus in patient-derived iPSC and one of these sgRNAs was also allele-specific for the E342K (G > A) point mutation, which could be used for monoallelic repair of heterozygous patient cell lines in the future. Moreover, we have demonstrated the usefulness of this *in cella* reporter assay for the testing of sgRNAs for two additional disease-related loci, such as the site of the V30M mutation in the TTR gene and the site of the D90A and R115G mutations in the SOD1 gene. Together, our work underlines that careful planning and choice of appropriate sgRNAs is essential for development of a successful and safe precision genome engineering approach.

Moreover, we demonstrate that the choice of an appropriate promoter can be crucial for successful genome engineering strategies, which involve selectable long flanking arm donors. While we were successful in getting correctly bi-allelic targeted clones with the mouse PGK promoter, we were unable to counter-select for excised clones after piggyBac transfection, most likely due to rapid silencing of the PGK promoter cassette in the transcriptionally inactive genomic locus. We have used a selectable long flanking arms donor construct previously published by Yusa and colleagues^10^,^11^ made available through Wellcome Trust Sanger and our results seemingly challenge previous protocols published with this construct. However, our data must not necessarily oppose the findings of Yusa *et al*., as we have used an entirely different iPSC cell line for our approach and it is known that vast epigenetic differences can exist between various iPSC cell lines, even when they have originated from the same reprogramming attempt^25^. Previously, we showed complete reprogramming and full genomic integrity in the hPi cell line we have used here^20^, but for future more in-depth investigations it could be interesting to directly compare the epigenetic configuration as well as expression levels of epigenetic modifier enzymes such as DNMTs and TETs in the cell lines used by Yusa and colleagues with our cell line. Of note, the PGK-based donor construct published by Kosuke Yusa has also been used by others in the mean time for efficient mono-allelic and allele-specific Cas9-based gene targeting of the *SERPINA1* locus^26^. However, despite commercial availability of piggyBac transposase and apparent simplicity of excision protocols, Smith and colleagues did not show data on selection cassette excision which would confirm the findings published by Yusa *et al*. in 2011. While can only speculate about the reasons behind this, it is possible that they faced similar challenges with piggyBac-based excision as well. Last but not least, as indicated in the troubleshooting section of the 2013 Nature Methods publication of Yusa *et al*. transposons can succumb to silencing in certain genomic regions, which can render efficient counter-selection impossible^11^. Our approach with the GC-rich CAG promoter presents a viable alternative for cases where region- or cell line-specific transgene silencing effects can be an issue. We demonstrate that this promoter is by far more silencing-resistant than the PGK promoter and that it can be used to achieve 100% counter-selection efficiency in bi-allelic targeted clones in a cell line where it was previously impossible to select for bi-allelic excised clones. However, the large nature of the newly designed selection/counter-selection cassette including the CAG promoter and a T2A-coupled fluorescence marker made efficient bi-allelic targeting impossible at first. In our lentiviral *in vitro* assay we found lower expression levels of the eGFP transgene in PGK-transduced cells than in CAG-transduced cells, which could have consequences for gene targeting. However, the total number of puromycin-resistant colonies we isolated when using the CAG construct was comparable to the number we had received when using the PGK promoter with the otherwise same protocol and same cell line as before (PGK: 10 colonies, CAG: 8, 13 and 12 colonies). Therefore we rejected the hypothesis that potentially lower expression levels of the puro*Δtk* cassette from the PGK promoter could have given a selective advantage to bi-allelic targeted clones in the PGK transfected cells. Meanwhile, it is much more likely that the more than 1800 bp larger insert had negative consequences for the HDR process itself and previous reports on HDR efficiencies with different insert sizes support this view^27^,^28^. Nevertheless, we achieved bi-allelic targeting at the *SERPINA1* locus using a dual-fluorescence selection strategy with a total efficiency of ~10%. We think however, that in the future this efficiency might be further improved by a more stringent gating strategy for FACS sorting combined with preparation of larger batches of transfected and puro-selected cells. Nevertheless, after piggyBac transfection and FIAU-selection, all viable clones showed bi-allelic excision and had a functionally corrected phenotype at the end of hepatic differentiation. Together, our report highlights the impact of an appropriate promoter for genome engineering approaches employing selection and counter-selection cassettes. We found that the CAG promoter meets the criteria for efficient modification of the transcriptionally silent *SERPINA1* locus in a cell line which was resistant to modification when using a more silencing-prone construct. Combining the large CAG promoter with a dual-fluorescence selection strategy we were able to achieve bi-allelic targeting and 100% counter-selection efficiency.

While our approach is a viable strategy for bi-allelic correction of a point-mutation, there is the option to omit plasmid donors and use ssODN donors instead. This method is elegant but it also comes with various disadvantages. First, the rate of correct incorporation of ssODNs is rather low and generally, hundreds of clones have to be analyzed. For example, Paquet and colleagues have used bar-coding of PCR-amplicons from single iPSC colonies followed by extensive bioinformatic analysis in order to identify HDR clones^15^. Moreover, it was demonstrated that inactivation of the PAM in an ssODN donor can prevent Cas9 nuclease from re-binding to the corrected genomic DNA, which improved overall HDR efficiency. However, with increasing distance from the to-be-introduced genomic modification to the PAM mutation, the chance of homozygous incorporation vastly decreased and dropped below 10% for distances of more than 15 bp^15^. This is also most likely the reason why the highest rates of bi-allelic modification were measured when sgRNAs which bind in close proximity to the intended mutation were used. However, there are many genomic loci where such favorable pre-conditions are not given, and moreover, silent PAM mutations may not always be possible. In such cases, approaches with long flanking arm plasmid donors may be more suitable, because chance of homozygous incorporation decreases in a linear manner from the genomic cutting site along the homology with the flanking arms in human iPSC^29^. Consequently, this would lead to a higher chance of co-incorporation of mutated PAM and intended modification even at larger distances in between. Second, insertion of larger DNA fragments can be achieved by plasmid donors, and has been applied for targeted correction of exon deletions in the Dystrophin gene in Duchenne muscular dystrophy patient cells^30^. Using an efficient selection/counter-selection approach like the one we describe here is generally applicable for such corrections as well and should be able to boost efficiencies in cases, where ssODNs are too short for targeted correction. Third, it remains to be investigated whether ssODNs or plasmid donors are generally more suitable for clinically-oriented gene editing approaches, as in-depth studies about potential remnants of DNA-donors in the genome and consequential safety-related questions are still pending. Currently, however, larger remnants of plasmid donors should be detectable by simple PCR methods whereas small fragments of ssODNs would be much harder to detect. For those reasons, we appraise that despite nowadays availability of somewhat efficient protocols for ssODN-based gene editing, plasmid donors do have certain advantages and may be a more favorable choice depending on the exact aim of the study. The approach we present here helps to increase efficiencies for bi-allelic editing when performing such plasmid donor-based genome engineering studies.

## Experimental Procedures

### Cloning and plasmids

A detailed description of all cloning procedures, used plasmids and primer sequences can be found in Supplementary Experimental Procedures and in Supplementary Table S4.

### *In vitro* Cas9 digest with tracrRNA and crRNA

M-AAT and Z-AAT targets were synthesized and inserted into the pEX-A vector (Eurofins Genomics, Ebersberg, Germany), as described in Supplementary Experimental Procedures and Supplementary Table S4. SpCas9 nuclease was produced and purified as described earlier^31^. Edit-R tracrRNA and synthetic crRNA were ordered from Dharmacon (GE Healthcare Life Sciences, Freiburg, Germany), where the crRNA was designed to target the DNA sequence 5′-GTGCTGACCATCGACAAGAA-3′. *In vitro* Cas9 digest was then performed based on a previously described protocol^31^. Briefly, tracrRNA, crRNA and spCas9 protein were pre-incubated in KGB buffer for 15 min at 25 °C and both plasmids were first linearized with PvuI and purified by gel electrophoresis. Subsequently, a 20 μl reaction mix was set up to achieve final concentrations of 10 nM for tracrRNA, 10 nM for crRNA, 5 nM for SpCas9 protein and 5 nM (~10 ng/μl) for the plasmids and incubated at 37 °C for 15 min.

### Production and titration of lentiviral vectors

All lentiviral vectors were produced by transient transfection of HEK293T cells using the standard CaCl_2_ method, as described previously^20^. 36 hours post transfection supernatants were harvested, filtrated through a 0.45 μm bottle-top filter (Sarstedt, Germany) and subjected to ultracentrifugation at 14,000 × g for 8 h. Pellets were re-suspended in ice-cold PBS, using 0.5% of the total supernatant volume (200x concentration) and frozen in 100 μl aliquots at −80 °C. Titers of lentiviral vectors CiG-MAAT-t-IP, CiG-ZAAT-t-IP, CiG-TTR-t-IP, CiG-SOD1-t-IP and Alb-Neo were determined by a dilution series transduction of HEK293T cells and subsequent multiplex qRT-PCR analysis of isolated genomic DNA for vector copy numbers using specific primer/probe sets for the WPRE element of the vectors and the genomic PTBP2 locus, as described previously^20^. Titers of lentiviral CGIP and PGIP vectors were determined by dilution series transduction followed by FACS analysis 72 h post transduction.

### Cell lines, transfections and transductions

Human H9 embryonic stem cells and the patient-derived iPSC cell line homozygous for Z-AAT (hPi) were published previously and cultivated according to standard protocols^20^. hPi-GFP corresponds to previously published hPi-s3, a cell clone derived from hPi constitutively expressing eGFP from a lentiviral CAG construct, joined to a miR-30-styled scramble shRNA^20^.

HEK reporter cells for Z-AAT, M-AAT, TTR and SOD1 targets were generated by transduction of HEK-293T cells at low MOI of 0.1 followed by 3 days of expansion and 6 days of selection using 2 μg/ml puromycin (Sigma-Aldrich #P7255). Transduced bulk populations were then further expanded without puromycin and seeded at 80,000 cells/well of 12-well for the reporter assay. The following day, reporter cells were transfected using branched polyethyleneimine (PEI, Sigma-Aldrich #408727). Per well of 12-well we mixed 0.5 μg hCas9 or hCas9_D10A + 0.25 μg of sgRNA + 0.5–2.5 μg of tGIP donor with 1.25 μl 0.1 mM PEI and added DMEM-GlutaMAX™ w/o additives to a total volume of 50 μl, followed by 10 min incubation at room temperature. In order to ensure constant concentration of total DNA in each experiment series, an appropriate amount of empty pCR2.1-TOPO plasmid was added to the transfection mix where needed. Next, we added 300 μl of DMEM-GlutaMAX™ with 12% FBS and replaced the cell culture medium with the transfection mix. Cells were incubated for 4 h before the transfection mix was replaced by DMEM-GlutaMAX™ with 10% FBS. 72 hours post transfection cells were washed with PBS and trypsinized for FACS analysis.

Transgenic human iPSC harboring either PGK-eGFP-IRES-puro (PGIP) or CAG-eGFP-IRES-puro (CGIP) were generated by lentiviral transduction of hPi cells at MOI of 50, based on a previously described protocol^20^. At day 3 post transduction, cells were selected using 1 μg/ml puromycin for two days and further expanded using 0.5 μg/ml puromycin. For the silencing assay, each bulk population was seeded onto six wells of a 6-well, where three were continuously kept on 0.5 μg/ml puromycin and in the other three puromycin was withdrawn for 7 days. Transduced hPi were then separated into single cells and stained with PE Mouse Anti-Human TRA-1–60 Antigen (BD Pharmingen #560884) for 45 min on ice and washed twice with PBS before analysis in a BD FACSCalibur™ (BD Biosciences). For calculation of percentage of transgene silencing, only TRA-1–60 positive cells were considered and the percentage of eGFP-negative cells from hiPSC kept on puromycin was subtracted from the cells kept off puromycin. In total, n = 4 separate bulk populations were generated and analyzed for each, CGIP and PGIP.

For gene editing of hPi cells, cells were grown to 80% confluence and treated with 10 μM ROCK inhibitor Y27632 one day before the starting of the procedure. Next day, colonies were washed with PBS and dissolved with a 1:1 mixture of Trypsin and PBS. Detached cells were filtered through a 70 μm cell strainer (BD Biosciences) directly into DMEM with 10% FBS for inactivation of Trypsin, centrifuged, resuspended in PBS and again filtered through a 70 μm cell strainer in order to get rid of remaining clumps. Finally, single cells were counted and either resuspended in Buffer R of the Neon^®^ Transfection Kit (Invitrogen) for electroporation or seeded on Matrigel™-coated dishes (Geltrex^®^, Thermo Fisher Scientific) for lipofection next day. For transfections of gene targeting experiments with a single donor construct, 2 × 10^6^ human iPSC were electroporated in 100 μl buffer R with 5 μg hCas9 or hCas9_D10A + 2.5 μg of either AAT_g1 sgRNA alone or AAT_g1 + AAT_g2 sgRNAs together + 10 μg of AAT_PB-PGKpuroTK or AAT-PB-CG2Ap*Δtk* donor plasmid with 2 × 20 ms pulses at 1000 V and immediately transferred into ice-cold hES medium with ROCK inhibitor, centrifuged, resuspended and counted. Viability after treatment was around 10% and 10,000 surviving cells were plated per well of a CF1γ-MEF coated 6-well in 2/3 MEF-conditioned 1/3 fresh hES medium with 10 μM ROCK inhibitor and 40 ng/ml bFGF (cond-hES). At day 3 post transfection, medium was replaced with cond-hES medium with 1 μg/ml puromycin for 2 days and cells were kept continuously on 0.5 μg/ml puromycin after that. For transfections of gene targeting experiments with two donor constructs, 80,000 cells/well were seeded in 36 wells of Matrigel™-coated 12-well plates in cond hES + RI. Next day, medium was replaced with 300 μl Opti-MEM^®^ (Gibco, Thermo Fisher Scientific) with 10 μM ROCK inhibitor and 40 ng/ml bFGF and cells were transfected using Lipofectamine LTX (Invitrogen, Thermo Fisher Scientific) as follows, per well of 12-well: in tube #1, 0.6 μg hCas9_D10A + 0.3 μg AAT_g1 + 0.3 μg AAT_g2 + 0.6 μg AAT-PB-CG2APtk + 0.6 μg AAT-PB-CD2APtk were mixed with Opti-MEM^®^ to a total volume of 47.6 μl and 2.4 μl PLUS reagent was added. In tube #2, 4.5 μl LTX + 45.5 μl Opti-MEM^®^ was incubated for 5 min at room temperature. Tube #1 and tube #2 were mixed and incubated for 10 more min before the 100 μl transfection was added per well. Medium was exchanged by cond-hES 4 h later, and cells were selected with puromycin from d3 post transfection as above. Two weeks post transfection cells were separated to single cells, which then were sorted for eGFP^+^ high, DsRed^+^ high and double positive fractions using a BD FACSAria Fusion cell sorter (BD Biosciences) and 2,000 sorted cells were plated each onto a CF1γ-MEF coated 10 cm dish in cond-hES. Single colonies were picked 2 weeks later and expanded for analysis. Using the same protocol as above, bi-allelic targeted cell lines hPi-#7, hPi-#8, hPi-#9, hPi-DG#5 and hPi-DG#22 were electroporated with 10 μg of pCMV-hyPBase^10^ for excision of the transgene. Clone hPi-DG#5 reacted in a hypersensitive manner to single cell separation and only few cells survived the treatment. The other clones we selected using 0.5 μM Fialuridine (FIAU, Sigma-Aldrich #SML0632) from day 4 to day 14 post transfection and arising colonies were half-picked onto two wells of a CF1γ-MEF coated 24-well. After 7 more days 0.5 μM puromycin was added to one of the wells and the other well was used to expand the FIAU-resistant, puro-sensitive sub-clones.

H9 human embryonic stem cells were obtained from WiCell (Madison, WI, USA). hPi human induced pluripotent stem cells were generated according to institutional guide lines after obtaining informed consent from the respective patient by the group of Dr. Axel Schambach at Hannover Medical School, Hannover, Germany and first described in Li *et al*.^32^ as iPSC line “AP-iPS-C1”, whereas they were fully characterized by our group in a subsequent publication^20^. HEK-293T cells were a kind gift from Dr. Axel Schambach at Hannover Medical School, Hannover, Germany. All methods involving the use of human iPSCs were carried out in accordance with institutional guidelines of Hannover Medical School and all experimental protocols involving the use of human ESCs were approved by the German Federal Authorities (RKI: AZ 3.04.02/0105).

### Lentiviral vector copy number (VCN) assay

Average numbers of lentiviral integrations were determined according to a qRT-PCR based method employing primers and probes specific for genomic PTBP2 and lentiviral WPRE, as described earlier^20^.

### PCRs for analysis of transgene insertion

All PCRs for analysis of integration of long flanking arm donor constructs were performed using Phusion Hot Start II DNA Polymerase with HF-buffer (Thermo Scientific #F549L) according to manufacturer’s procedures. Melting temperatures of primers were calculated using the Tm calculator on the Thermo Scientific homepage (https://www.thermofisher.com/) and a 6-step gradient PCR was performed from the lowest oligo’s Tm −2 °C to the highest oligo’s Tm +6 °C in order to determine the optimal temperature yielding specific products. All sequences of PCR primers with their corresponding applications and applied annealing temperatures are given in Supplementary Table S4.

### Hepatic differentiation of human iPSC

Hepatic differentiation of pluripotent stem cells was performed based on a previously described protocol^20^, with the slight modification that a lentiviral Alb-Neo selection cassette without fluorescence was used, in order to allow for easier co-staining for polymeric and total AAT.

### Neutrophil elastase inhibition assay

Inhibition of neutrophil elastase in supernatants of hepatic differentiated hPi cells was assayed by Neutrophil Elastase Inhibitor Screening Kit (Abcam #ab118971) according to manufacturer’s procedures and measured on a Tecan Infinite M200 plate reader at Ex/Em = 400/505 nm.

### Immunofluorescence analysis

Human iPSC differentiated on Corning^®^ Matrigel^®^-coated dishes for 17 days were fixed using 4% PFA for 20 min at room temperature, washed 3x using PBS, permeabilized with PBS 0,5% Tween 0,2% Triton-X 100, 0,2% Igepal for 20 min, blocked with PBS 0,1% Tween, 5% filtered donkey serum for 1 h and incubated with primary antibodies for total AAT, goat anti-human AAT A80–122 A (Bethyl, Montgomery TX, USA) diluted 1:500 and for polymeric AAT, mouse anti-human AAT clone 2C1 (Hycult Biotech, Uden, Netherlands) diluted 1:50 in PBS 0,1% Tween 1% filtered donkey serum at 4 °C overnight. Next day, cells were washed 3x with PBS and incubated with secondary antibodies donkey-α-goat Alexa Fluor^®^ 568 (A-11057, Thermo Fisher Scientific, Germany) diluted 1:2000 and donkey-α-mouse Alexa Fluor^®^ 488 (715-545-150, Jackson ImmunoResearch, West Grove, PA, USA) diluted 1:400 in PBS 0,1% Tween 1% filtered donkey serum for 1 h at room temperature.

### Western Blots

SDS-PAGE Western blot was performed based on a previously described protocol^20^. Mouse anti-AAT monoclonal antibody 2B12 (Thermo Scientific #MA5-15521) was used at dilution 1:500 and mouse anti-vinculin (clone hVIN-1, Sigma #V9131) was used at a dilution 1:1000. Secondary antibody donkey IgG anti-mouse IgG (H + L)-HRPO (Dianova #715-035-150, for SDS-PAGE blot) and donkey IgG anti goat IgG (H + L)-HPRO (Dianova #705-035-147, for native blot) were diluted 1:2000. Native gel electrophoresis was performed based on a previously published protocol^33^, using a 4–15% gradient Mini-PROTEAN® TGX™ Precast Gel (Biorad #4561084) and blotted overnight onto a PVDF membrane at 200 mA at 4 °C. The next day, membrane was incubated with goat anti-AAT polyclonal antibody A80–122 A antibody (Bethyl, Montgomery TX, USA) diluted 1:500 in TBS-T 0,5% (w/v) milk powder.

### Southern blots

Southern blots were performed using an optimized protocol based on a previously published method^34^. Briefly, probes #1 and #2 were PCR amplified using Phusion HSII polymerase and purified over a 0.8% agarose gel and sequenced (see Supplementary Table S4 for primers and sequences). 10 μg genomic DNA was digested using 50U of either EcoRI (NEB #R0101S) or PagI (BspHI) (Fermentas #ER1281) enzyme in a total volume of 50 μl for 6 h at 37 °C and separated on a 0.8% agarose gel at 24 V overnight. DNA was then transferred to Biodyne B nylon membrane (Thermo Fisher Scientific #77016) by capillary force transfer in SSC buffer. 100 ng of DNA probes were labeled with 5 μl α^32^ PdCTP using 5 U/μl Klenow fragment (3000 Ci/mmol) in a total volume of 50 μl and used for hybridization. Washed membranes were then used for overnight exposure of Storage Phosphor Screens “BAS-IP MS 2040 E Multipurpose Standard” (GE Healthcare Life Sciences #28-9564-74) which were analyzed next day using the Amersham Molecular Devices Storm 820 Phosphorimager (GE Healthcare Life Sciences).

## Additional Information

**How to cite this article**: Eggenschwiler, R. *et al*. Improved bi-allelic modification of a transcriptionally silent locus in patient-derived iPSC by Cas9 nickase. *Sci. Rep.* **6**, 38198; doi: 10.1038/srep38198 (2016).

**Publisher's note:** Springer Nature remains neutral with regard to jurisdictional claims in published maps and institutional affiliations.

## Supplementary Material

Supplementary Information
