# Supplementary material for: Improved bi-allelic modification of a transcriptionally silent locus in patient-derived iPSC by Cas9 nickase
Source: Sci Rep. 2016 Dec 2;6:38198. doi: 10.1038/srep38198 (PMC5133597; doi:10.1038/srep38198)
Supplement: Supplementary Information [file srep38198-s1.pdf]

Supplementary Information

Improved bi-allelic modification of a transcriptionally silent locus in patient-derived iPSC by Cas9 nickase

Reto Eggenschwiler*,[1,2] Mohsen Moslem,[1,2,3] Mariane Serra Fráguas,[1,2,4] Melanie Galla[5], Oliver Papp[1,2], Maximilian Naujock[6,7], Ines Fonfara,[8,9] Ingrid Gensch,[1,2] Annabell Wähner,[1,2] Abbas Beh-Pajooh,[1,2] Claudio Mussolino,[10,11,12] Marcel Tauscher[13], Doris Steinemann[13], Florian Wegner[6], Susanne Petri[6], Axel Schambach[5], Emmanuelle Charpentier,[8,9] Toni Cathomen,[10,11,12]
Tobias Cantz*,[1,2,14]

[1]Research Group Translational Hepatology and Stem Cell Biology, Cluster of Excellence REBIRTH, Hannover Medical School, Hannover, 30625, Germany
[2]Department of Gastroenterology, Hepatology and Endocrinology, Hannover Medical School, Hannover, 30362, Germany
[3]present address: Department of Neuroscience, Karolinska Institute, Stockholm, 171 77, Sweden
[4]present address: Vascular Biology Division, Department of Medical Biochemistry and Biophysics (MBB), Karolinska Institute, Stockholm, 171 77, Sweden
[5]Institute of Experimental Hematology, Cluster of Excellence REBIRTH, Hannover Medical School, Hannover, 30625, Germany
[6]Department of Neurology, Hannover Medical School, Hannover, 30625, Germany
[7]present address: Boehringer Ingelheim Pharma GmbH & Co. KG, CNS Diseases Research, Biberach, 88400, Germany
[8]Max Planck Institute for Infection Biology, Department of Regulation in Infection Biology, Berlin, 10117, Germany
[9]The Laboratory for Molecular Infection Medicine Sweden (MIMS), Umeå Centre for Microbial Research (UCMR), Department of Molecular Biology, Umeå University, Umeå, 90187, Sweden
[10]Center for Chronic Immunodeficiency, Medical Center - University of Freiburg, Freiburg, 79106, Germany
[11]Institute for Cell and Gene Therapy, Medical Center - University of Freiburg, Freiburg, 79106, Germany
[12]Faculty of Medicine, University of Freiburg, Freiburg, 79106, Germany
[13]Institute of Human Genetics, Hannover Medical School, Hannover, 30625, Germany
[14]Max Planck Institute for Molecular Biomedicine, Cell and Developmental Biology, Münster, 48149, Germany

Table of Contents

Supplementary Figure **S1**	page 2
Supplementary Table **S2**	page 4
Supplementary Figure **S3**	page 5
Supplementary Table **S4**	page 6
Supplementary Figure **S5**	page 8
Supplementary Figure **S6**	page 11
Supplementary Table **S7**	page 12
Supplementary Figure **S8**	page 26
Supplementary Experimental Procedures	page 28

Supplementary Figure S1: Allele-specific nicking, efficiencies of different nickases and analysis of sgRNAs for other disease-related loci

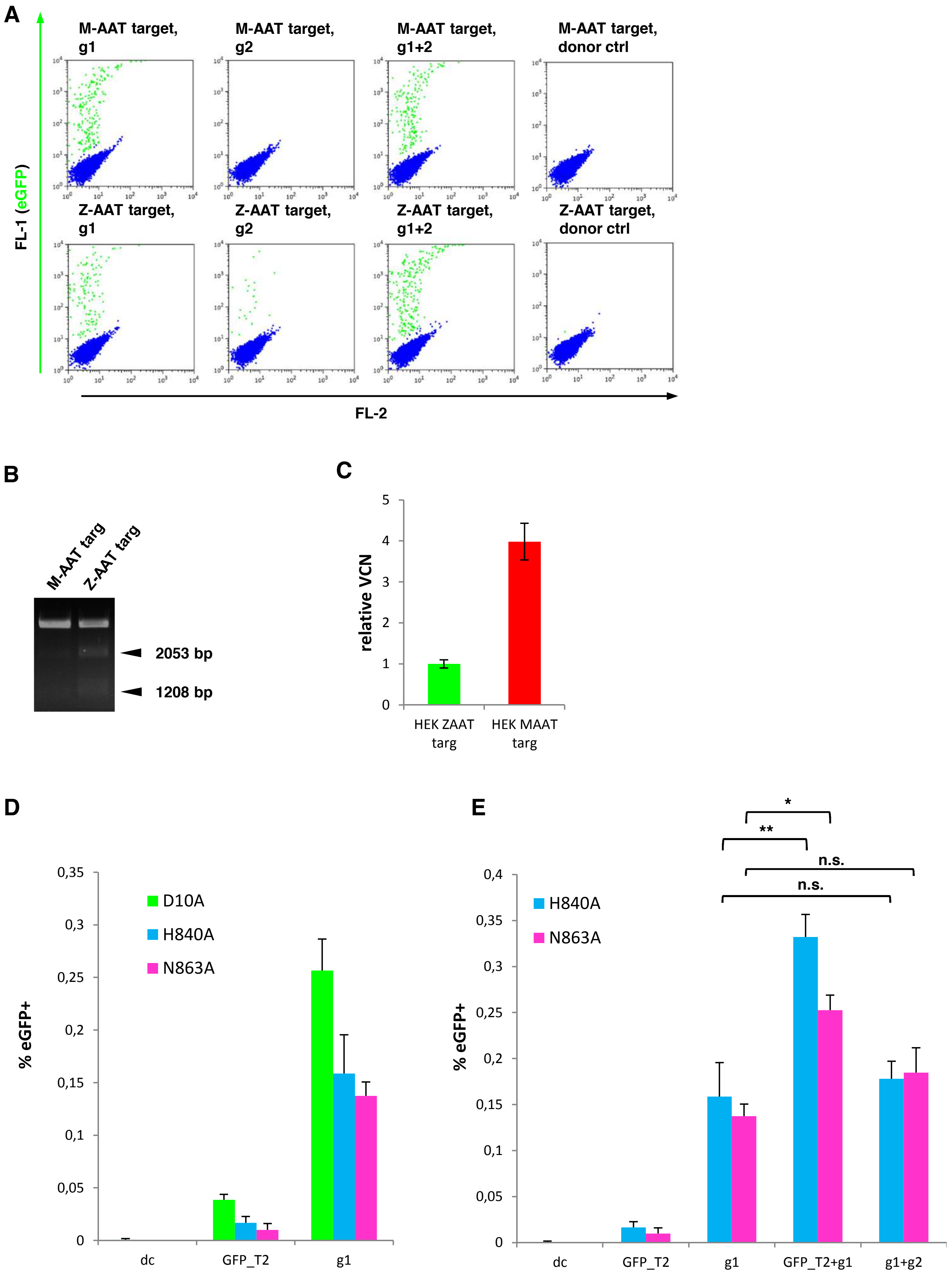

F

Targeting of TTR sequence in proximity of V30M mutation , which causes Familial Amyloid Polyneuropathy (FAP)

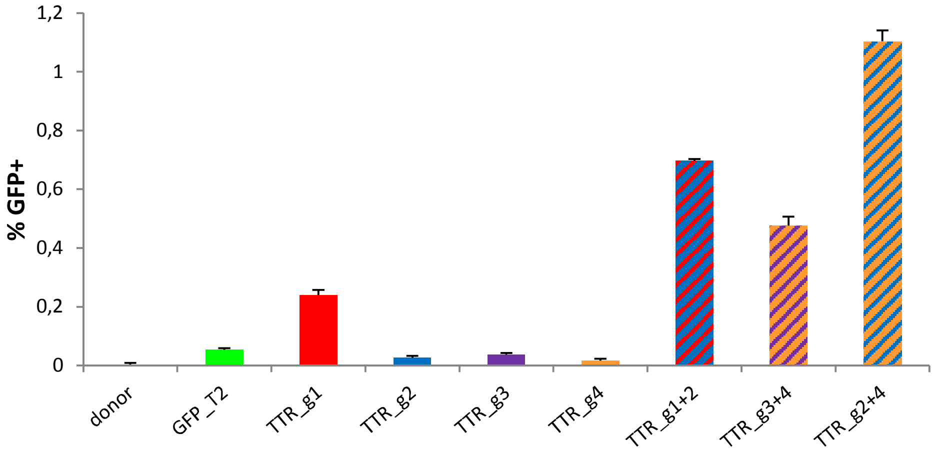

G

Targeting of SOD1 sequence in proximity of D90A and R115G mutations , which cause Amyotrophic Lateral Sclerosis (ALS)

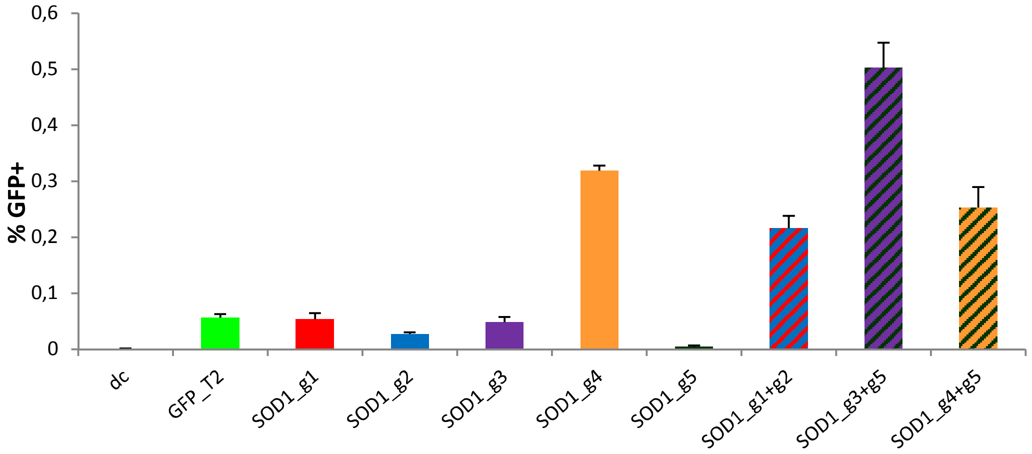

Supplementary Figure S1: Allele-specific nicking, efficiencies of different nickases and analysis of sgRNAs for other disease-related loci (A) Representative FACS blots of HEK M-AAT and Z-AAT reporter cells transfected with tGiP donor + Cas9_D10A + AAT_g1 gRNA (g1), AAT_g2 gRNA (g2) or AAT_g1 + AAT_g2 gRNAs (g1+2). 50'000 events gated from FSC/SSC are displayed. **(B)** *In vitro* Cas9 digest of plasmids containing M-AAT or Z-AAT target sequences using tracrRNA + crRNA_AAT_g2. **(C)** QRT-PCR-based analysis of relative lentiviral vector copy numbers in M-AAT and Z-AAT reporter cell lines. **(D)** FACS analysis of HEK Z-AAT reporter cells transfected with different nickases Cas9_D10A, Cas9_H840A and Cas9_N863A. **(E)** FACS analysis of HEK Z-AAT reporter cells transfected with HNH-deficient nickases Cas9_H840A or Cas9_N863A and different sgRNAs and sgRNA combinations. Double nicking with AAT_g1 gRNA and GFP_T2 gRNA results in a 5' overhang, increasing overall HDR efficiency, whereas double nicking with AAT_g1 gRNA and AAT_g2 gRNA results in a 3' overhang and no significant change in HDR efficiency. dc: donor control = cells transfected with Cas9_D10A and tGIP donor only. **(F)** FACS analysis of HEK TTR reporter cells transfected with Cas9_D10A, tGiP donor and different TTR sgRNAs. **(G)** FACS analysis of HEK SOD1 reporter cells transfected with Cas9_D10A, tGiP donor and different SOD1 sgRNAs. dc = donor control (Cas9_D10A + tGiP only), GFP_T2 = sgRNA binding to eGFP upstream of the target sequence, serving as internal reference.

Data are represented as mean ± SD and statistical analysis was performed by two-tailed student's t-test where * = $p < 0.05$, ** = $p < 0.01$, n.s. = not significant, n = 3 biological replicates.

Supplementary Table S2: top five potential off-targets of AAT sgRNAs

AAT_g1 sgRNA:

sequence	score	mismatches	UCSC gene	locus
GCACACCATCATGCACGGCCCAG	1,4	3MMs [6:8:10]		chr7:-152171049
CCCCATCCTGATGCACGGCCCGG	0,8	4MMs [1:3:6:10]	NR_038080	chr17:-79277406
GAGCTGCCTTCTGCACGGCCAGG	0,8	4MMs [2:3:5:11]		chr1:-45138716
CCACAGCATGCTGCACGGCCAGG	0,7	4MMs [1:8:10:11]	NM_001089	chr16:+2369799
CCACAGCCTTCTGCACTGCCAAG	0,7	3MMs [1:11:17]		chr2:-101478981
**off-target score**	**4,4**			

AAT_g2 sgRNA:

sequence	score	mismatches	UCSC gene	locus	comments
GTGCTGACCATCGACGAGAAAGG	17,2	1MMs [16]	NM_001002235	chr14:-94844958	(SERPINA1)
GAGCTGACCATCTACAAGAAGAG	3,6	2MMs [2:13]		chr1:-196503098	
GTGGTGACCATCAACAAGAAGAG	3,1	2MMs [4:13]	NM_001166434	chr3:+52824768	
GTGGTGAGCATGGACAAGAATGG	1,3	3MMs [4:8:12]		chr16:+22795769	
ATGCTGGCCATGGACAAGAAAAG	1	3MMs [1:7:12]		chr2:+53247163	
TTTCTGAACATCTACAAGAAAAG	0,6	4MMs [1:3:8:13]		chr4:+171459021	
	26,8				
	-17,2	(SERPINA1)			
**off-target score**	**9,6**				

AAT_g3 sgRNA:

sequence	score	mismatches	UCSC gene	locus	comments
TTCAGTCCCTTTCTCGTCGATGG	26,8	1MMs [15]	NM_001002235	chr14:+94844931	(SERPINA1)
CTCTTTCCCGTTCTTGTCGAAAG	1,3	4MMs [1:4:5:10]		chr1:-161015912	
TTCTTTCCCTTTCTTGTCTAAAG	1,0	3MMs [4:5:19]		chr9:+84446321	
AACATTCCCTCTCTTGTCGAGGG	0,8	4MMs [1:2:5:11]		chr2:+172685105	
TTTTGTCCCTTTCTTGTGGACAG	0,6	3MMs [3:4:18]		chr8:-26517054	
ATCAGTCCCTGTCTTGTCAAGAG	0,6	3MMs [1:11:19]		chr6:-78348659	
	30,5				
	-26,8	(SERPINA1)			
**off-target score**	**3,7**				

AAT_g4 sgRNA:

sequence	score	mismatches	UCSC gene	locus
GAAAGGGAGTGAAGCTGCTGGGG	61,1	1MMs [9]		chr2:+116934446
GGGAGGGACAGAAGCTGCTGCAG	2,4	3MMs [2:3:10]		chr7:-31097292
AAAGTGGACTGAAGCTGCTGCAG	2,4	3MMs [1:4:5]		chr12:-120809699
GAAGAGGACAGAAGCTGCTGAGG	2,3	3MMs [4:5:10]		chrX:+74959542
AAAAGGCACAGAAGCTGCTGAGG	1,7	3MMs [1:7:10]		chrX:-150553515
**off-target score**	**69,9**			

Supplementary Figure S3: FACS controls for silencing studies and analysis of puromycin selected clones generated with CAG donor construct

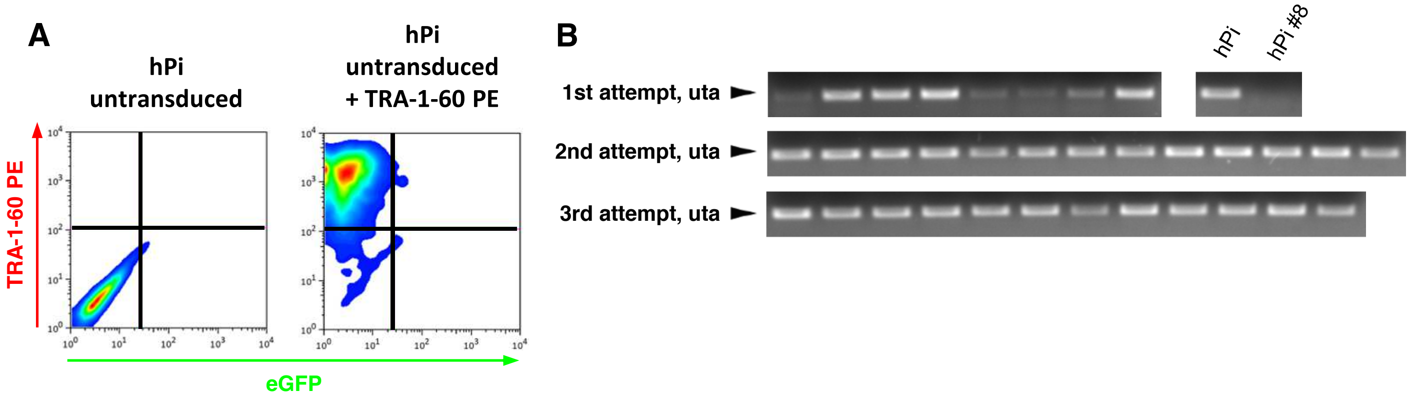

Supplementary Figure S3: FACS controls for silencing studies and analysis of puromycin selected clones generated with CAG donor construct (A) FACS controls of TRA-1-60 PE-stained and unstained hPi for gating of transgene silencing analysis. **(B)** PCR analysis for the untargeted *SERPINA1*-allele (uta) in AAT-PB-CG2Ap*Δtk* + Cas9_D10A + AAT_g1 + AAT_g2 transfected and puromycin-selected clonal cell lines. In 3 separate rounds and a total of 33 clones, no bi-allelic modifications were detected.

Supplementary Table S4: Cloning primers, cloned sequences and PCR primers

cloning primers

primer name	sequence
PGK ClaI for	GGTATCGATAAGCTTGATATCGAATAATTC
PGK XhoI rev	TTACTCGAGGGTTTAGTTCCTCACCTTGTCGTATTATAC
T2A BsrGI for	TTTTGTACAAGGGCTCCGGAGAGGGCCGG
T2A Tth111I rev	TTAGACGTCGTCGCGGGTGGCGAGGCGCACCGTGGGCTTGTACTCGGTCATGGAGGGGCCGG GGTTCTCCTC
DsRed AgeI for	TACCGTCGACGGATCCACCGGTC
DsRed SexAI mut rev	TCTTGGCCATGTAGATGGACTTGAACTCCAC**g**AGGTAGTGGCCGCCG
DsRed + YK rev	TTATGTACAGGAACAGGTGGTGGCGGC
CMV NheI for	TATGCTAGCGACATTGATTATTGACTAGTTATTAATAGTAATC
CMV MluI rev	AAAACGCGTGACCATGGTAATAGCGATGACTAATAC
CBA AscI for	AAAGGCGCGCCGAGGTGAGCCCCACGTTCTG
CBA PciI rev	AAAACATGTTGCCAAAATGATGAGACAGCAC
PGK AgeI for	CATCCACCGGTAGGCGCCAACCG
puro change RsrII rev	TTTCGGTCCGGGTCGACGGTGTGGCGCGTGGCGGGGTAGTCGGCGAACGCGGCGGCGAGGG TGCG**c**ACGGCCC

cloned sequences

sequence name	sequence
ZAAT target sequence	CCAGGCCGTGCATAAGGCTGTGCTGACCATCGAC**a**AGAAAGGGACTGAAGCTGCTGGGG*
MAAT target sequence	CCAGGCCGTGCATAAGGCTGTGCTGACCATCGAC**g**AGAAAGGGACTGAAGCTGCTGGGG
AAT_g1 guide sequence	G-CACAGCCTTATGCACGGCC
AAT_g2 guide sequence	G-TGCTGACCATCGACAAGAA
AAT_g3 guide sequence	G-TCAGTCCCTTTCTTGTCGA
AAT_g4 guide sequence	G-AAAGGGACTGAAGCTGCTG
	*: SNP rs28929474 (E342K)
TTR target sequence	CCCAGGGCACCGGTGAATCCAAGTGTCCTCTGATGGTCAAAGTTCTAGATGCTGTCCGAGGCA GTCCTGCCATCAATGTGGCC**a**TGCATGTGTTCAGAAAGGCTGCTGATGACACCTGGGAGCCATT TGCCTCTGGGCGCGCC*
TTR_g1 guide sequence	G-CTTGGATTCACCGGTGCCC
TTR_g2 guide sequence	G-AAGTTCTAGATGCTGTCCG
TTR_g3 guide sequence	G-GCATGGCCACATTGATGGC
TTR_g4 guide sequence	G-CCTGGGAGCCATTTGCCTC
	*: SNP rs28933979 (V30M)
SOD1 target sequence	ACCGGTCCTAATCCATCTGATGCTTTTTCATTATTAGGCATGTTGGAGACTTGGGCAATGTGACT GCTG**c**CAAAGATGGTGTGGCCGATGTGTCTATTGAAGATTCTGTGATCTCACTCTCAGGAGACC ATTGCATCATTGGC**g**GCACACTGGGGCGCGCC**
SOD1_g1 guide sequence	G-AAAAAGCATCAGATGGATT
SOD1_g2 guide sequence	G-ACTGCTGCCAAAGATGGTG
SOD1_g3 guide sequence	G-GAATCTTCAATAGACACAT
SOD1_g4 guide sequence	G-TGTGCCGCCAATGATGCAA
SOD1_g5 guide sequence	G-TGCATCATTGGCGGCACAC
	*: SNP rs80265967 (D90A); *: R115G

PCR primers for integration detection and southern blot probes

primer name	sequence	annealing (°C)	application
left AAT homol for	CAAAGCCGAGTTCCCATCTTAG	60 / 65.6	Fig. 2B, 2C, 3F, S5C / 3F (full length)
left AAT homol rev	GCGACTGAGATGTCCTAAATGCAC	60	Fig. 2B, 2C, 3F, S5C
right AAT homol for	TCTATGGCTTCTGAGGCGGAAAG	65.6	Fig. 2B, 2C, 3F, S5C
right AAT homol rev #3	TTCCTGGCTGCTGTGTCCTTTAG	65.6	Fig. 2B, 2C, 3F, S5C
det gen A1AT for	GTG TCC ACG TGA GCC TTG CTC	60	Fig. 2B, 2C, 3E, 3F, S3B, S5B
det gen A1AT rev	GTT TGT TGA ACT TGA CCT CGG	60	Fig. 2B, 2C, 3E, 3F, S3B, S5B
CAG end for	TCCTGGGCAACGTGCTGGTTATTG	69.6 / 66.5	Fig S5E (eGFP) / S5E (DsRedEx)
eGFP_det rev	TCACCTTGATGCCGTTCTTC	69.6	Fig S5E
DsRedEx rev	CGCGCTCGTACTGCTCCACG	66.5	Fig S5E
AAT_sb_left for	ATTCGTGATTATGCCCATGCCC	65.4	southern blot probe #1
AAT_sb_left rev	ATGGGAACTCGGCTTTGGTTT	65.4	southern blot probe #1
AAT_sb_right for	GCCATTGCAAGGACTCCTCA	65.4	southern blot probe #2
AAT_sb_right rev	TTCCTGGTAGAGACGGAGGG	65.4	southern blot probe #2

southern blot probes

probe #1 (549 bp)

ATTCGTGATTATGCCCATGCCCTGCTGATCTAGTTCGTTTTGTACACTGTAAAACCAAGATGAAA
ATACAAAAGGTGTCGGGTTCATAATAGGAATCGAGGCTGGAATTTCTCTGTTCCATGCCAGCAC
CTCCTGAGGTCTCTGCTCCAGGGGTTGAGAAAGAACAAAGAGGCTGAGAGGGTAACGGATCAG
AGAGCCCAGAGCCAAGCTGCCCGCTCACACCAGACCCTGCTCAGGGTGGCATTGTCTCCCCAT
GGAAAACCAGAGAGGAGCACTCAGCCTGGTGTGGTCACTCTTCTCTTATCCACTAAACGGTTGT
CACTGGGCACTGCCACCAGCCCCGTGTTTCTCTGGGTGTAGGGCCCTGGGGATGTTACAGGCT
GGGGGCCAGGTGACCCAACACTACAGGGCAAGATGAGACAGGCTTCCAGGACACCTAGAATAT
CAGAGGAGGTGGCATTTCAAGCTTTTGTGATTCATTCGATGTTAACATTCTTTGACTCAATGTAG
AAGAGCTAAAAGTAGAACAAACCAAAGCCGAGTTCCCAT

probe #2 (317 bp)

GCCATTGCAAGGACTCCTCAGCTATGGGAGAGGAAGCACATCACCCATTGACCCCCGCAACCC
CTCCCTTTCCTCC**c**CTGAGTCCCGACTGGGGCCACATGCAGCCTGACTTCTTTGTGCCTGTTGC
TGTCCCTGCAGTCTTCAGAGGGCCACCGCAGCTCCAGTGCCACGGCAGGAGGCTGTTCCTGAA
TAGCCCCTGTGGTAAGGGCCAGGAGAGTCCTTCCATCCTCCAAGGCCCTGCTAAAGGACACAG
CAGCCAGGAAGTCCCCTGGGCCCCTAGCTGAAGGACAGCCTGCTCCCTCCGTCTCTACCAGGA
A*

*: SNP rs1243166

off-target analysis PCR primers

primer name	sequence	annealing (°C)	genomic off-target site
AAT_g1_OT1 for	TTGTTTACCAGTCCCCAGGC	62.2	
AAT_g1_OT1 rev	TCAAGGCAAGGACTGTCGTC	62.2	GCACACCATCATGCACGGCCCAG
AAT_g1_OT2 for	TGGAAAATACCCTGTGGCCC	62.2	
AAT_g1_OT2 rev	TTTGAGGCTTTCCCGGTAGG	62.2	CCCCATCCTGATGCACGGCCCGG
AAT_g1_OT3 for	TTCCCAGATCCCTCCCGTAG	62.2	
AAT_g1_OT3 rev	GAGGGAGGCAGGACAAAACA	62.2	GAGCTGCCTTCTGCACGGCCAGG
AAT_g1_OT4 for	GACAGCGCGGTTTCTAGAGT	62.2	
AAT_g1_OT4 rev	TGTCAGTGTGTGGTGCGTTT	62.2	CCACAGCATGCTGCACGGCCAGG
AAT_g1_OT5 for	CCCAATGCGTGTTTCAGGTG	62.2	
AAT_g1_OT5 rev	CTGATCAGACCTCGGAAGGG	62.2	CCACAGCCTTCTGCACTGCCAAG
AAT_g2_OT1 for	TGTGGTTTCCGAGGTAGTGC	62.2	
AAT_g2_OT1 rev	CTCAGCTTGGGTGCAGAAGT	62.2	GAGCTGACCATCTACAAGAAGAG
AAT_g2_OT2 for	AAGGGCAGCTGAATGGAGAG	62.2	
AAT_g2_OT2 rev	ATCCGATGACTGTCCAGCAC	62.2	GTGGTGACCATCAACAAGAAGAG
AAT_g2_OT3 for	CATGCCAAAAATGCGAAGCAG	62.2	
AAT_g2_OT3 rev	GGCGCGGTCCTGTAATTTATC	62.2	GTGGTGAGCATGGACAAGAATGG
AAT_g2_OT4 for	ACCAAGTAGCAAGCTGACCA	62.2	
AAT_g2_OT4 rev	ACAATCACATAACATGGGACTGAAT	62.2	ATGCTGGCCATGGACAAGAAAAG
AAT_g2_OT5 for	ATGACAACTTTCACTGTCGCA	62.2	
AAT_g2_OT5 rev	TCACCCACAGAACGTAAGCA	62.2	TTTCTGAACATCTACAAGAAAAG

Supplementary Figure S5: Extended analysis of targeted and gene corrected clones

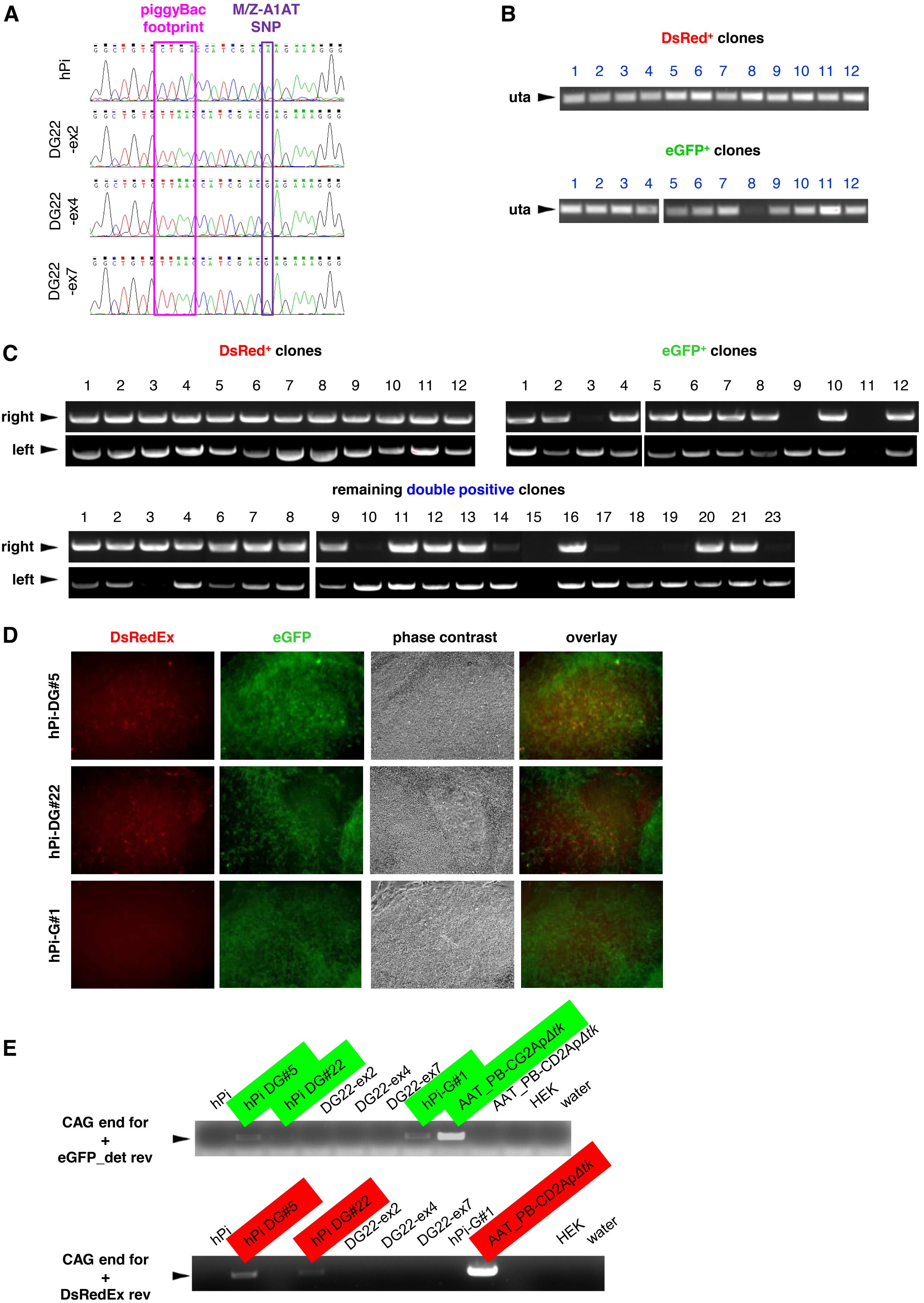

F uta: untargeted and/or excised alleles: 9596 bp (EcoRI), 8796 bp (BspHI)

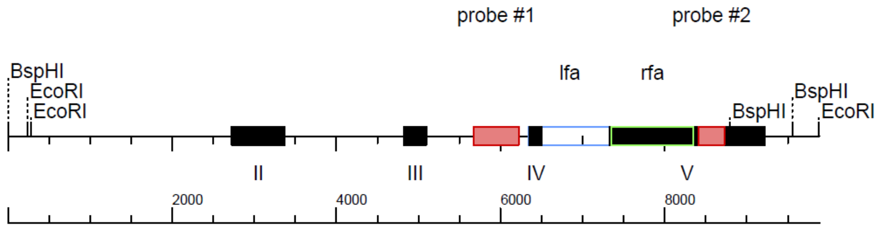

ta: targeted alleles, BspHI digest: 13860 bp (eGFP), 13824 bp (DsRedEx)

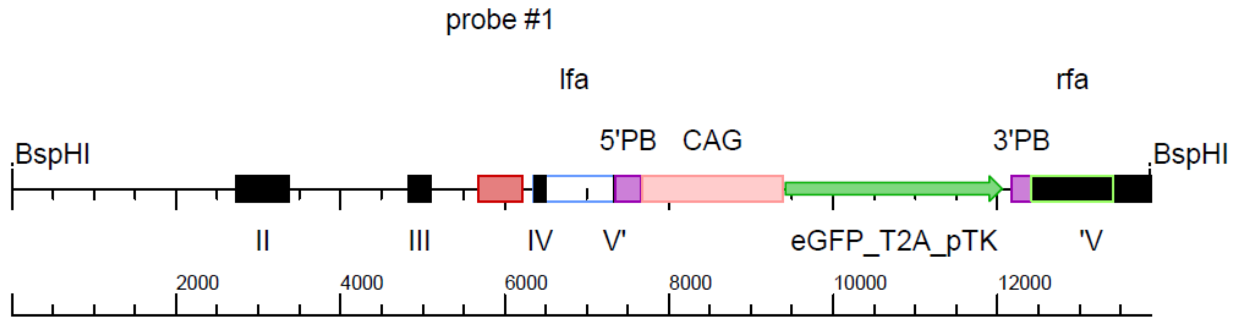

ta: targeted alleles, EcoRI digest: 5552 bp (eGFP), 5516 bp (DsRedEx)

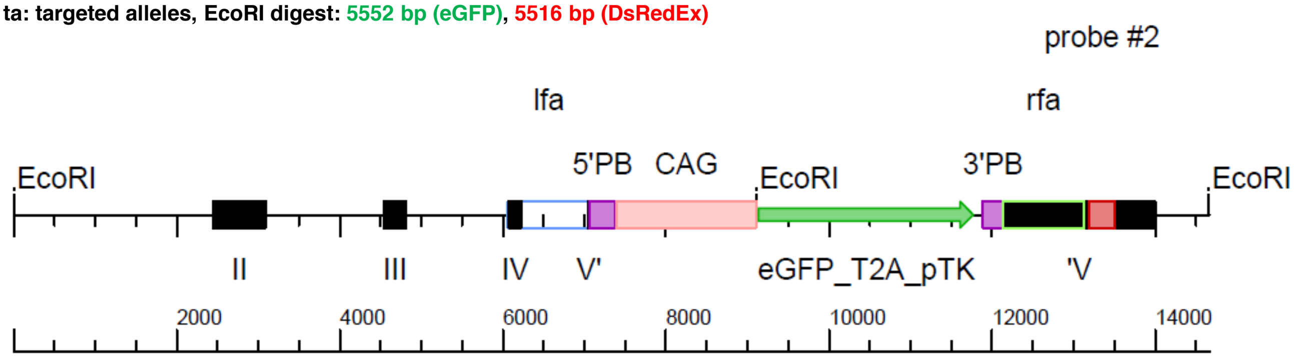

G EcoRI digest, probe#2

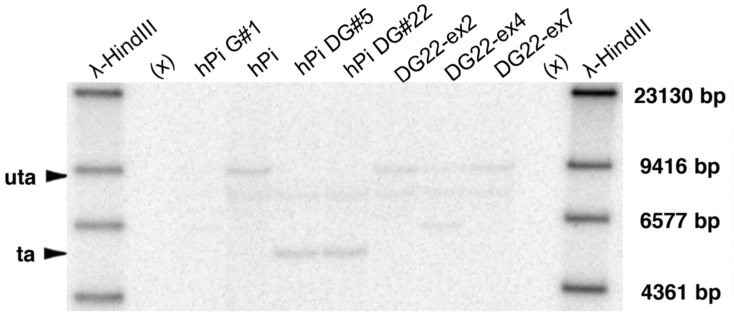

H BspHI digest, probe#1

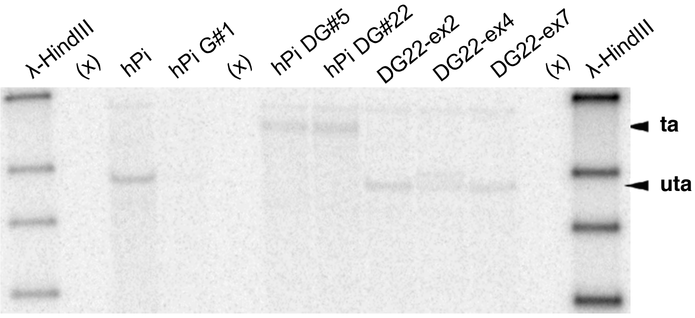

J EcoRI digest

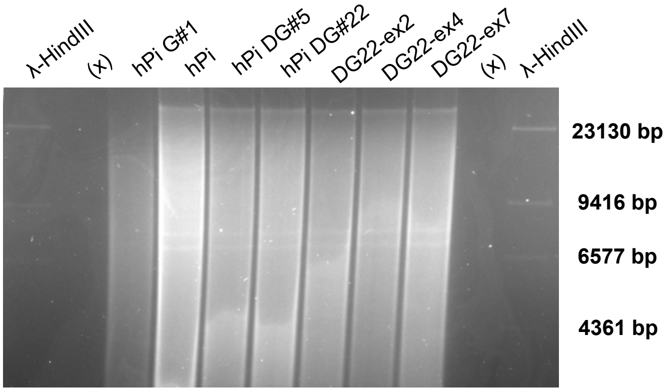

K BspHI digest

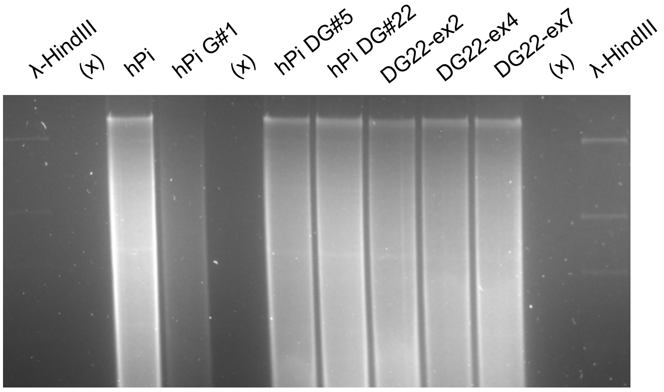

Supplementary Figure S5: Extended analysis of targeted and gene corrected clones (A) Sequencing of gene-corrected hPi-derived cell lines. PiggyBac footprint remains at a site in Exon V where the CTG-codon for leucin is followed by an A, leading to synonymous substitution of the codon. **(B)** PCR analysis for the untargeted *SERPINA1*-allele in sorted eGFP+ and DsRedEx+ single positive clonal cell lines **(C)** PCR analysis of clones isolated from sorted cell populations for integration of left and right homology flanking arms. **(D)** Fluorescence images depicting eGFP and DsRedEx signal in mono- and bi-allelic targeted hPi cells. hPi-G#1 were sorted from eGFP single positive fraction and are targeted on one allele, whereas hPi-DG#5 and hPi-DG#22 were sorted from double-positive fraction and are targeted on both alleles. **(E)** PCR analysis for detection of eGFP and DsRedEx in bi-allelic targeted and excised cell lines, as well as mono-allelic targeted hPi-G#1. hPi and an HEK239-T served as negative whereas diluted donor plasmids served as positive controls. **(F)** Setup of Southern blot for detection of donor plasmid integration and excision: gDNA digested with BspHI enzyme was probed with a 549 bp PCR fragment matching upstream of the left flanking arm (probe #1) and gDNA digested with EcoRI enzyme was probed with a 317 bp PCR fragment matching downstream of the right flanking arm (probe #2). lfa = left flanking arm (blue frame); rfa = right flanking arm (green frame); II, III, IV, V = human *SERPINA1* Exons (black boxes); 5'PB, 3'PB = piggyBac recognition sites (purple); CAG = CAG enhancer/promoter (rose); eGFP_T2A_pTK = fluorescence + selection / counter-selection cassette (green arrow). **(G)** Southern blot with EcoRI digested gDNA hybridized with probe #2. **(H)** Southern blot with BspHI digested gDNA hybridized with probe #1. **(J, K)** Agarose-gel images of Southern blots from Fig. S5G and S5H, showing successful digest and quality of gDNA.
λ-HindIII = Lambda DNA digested with HindIII marker; (x) = empty lane; ta = targeted allele; uta = untargeted allele.

Supplementary Figure S6: Extended functional characterization of bi-allelic targeted and bi-allelic corrected clones.

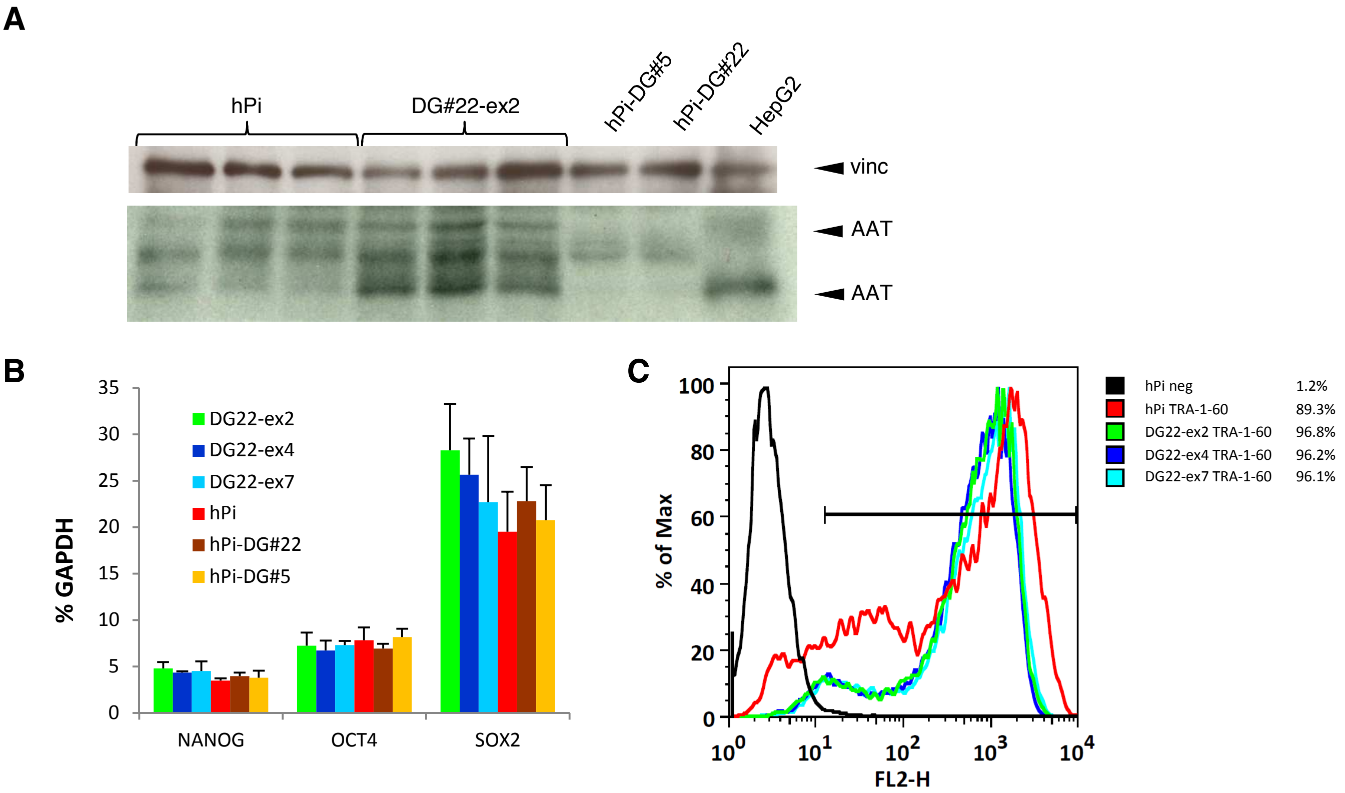

Supplementary Figure S6: Extended functional characterization of bi-allelic targeted and bi-allelic corrected clones (A) Western blot for total human AAT in d17 hepatic differentiated iPSC using mouse monoclonal 2B12 antibody. Differentiation of parental hPi and gene-corrected DG22-ex2. hPi-DG#5 and hPi-DG#22 served as negative whereas HepG2 served as positive control. **(B)** Taqman qRT-PCR expression-analysis of pluripotency markers NANOG**,** OCT4 and SOX2 in undifferentiated hPi, targeted hPi-DG#5 and hPi-DG#22 and gene-corrected DG22-ex2, DG22-ex4 and DG22-ex7. **(C)** FACS analysis for of hPi and gene corrected cell lines stained with α-TRA-1-60 PE-conjugated antibody.
Data in Fig. S6D are represented as mean ± SD with n = 3 biological replicates.

Genome: hg19
Aberration Algorithm: ADM-2
Threshold: 7.0
Fuzzy Zero: ON
GC Correction: ON
Window Size: 2Kb
Centralization (legacy): ON
Centralization (legacy) Bin Size: 10
Centralization (legacy) Threshold: 7.0
Diploid Peak Centralization: ON
Manually Reassign Peaks: OFF
Combine Replicates (Intra Array): ON
Combine Replicates (Inter Array): ON
Inter Array Group By: Amt Cy3 used(ug)
Array Level Filter: NONE
Aberration Filter: (Minimum Number of Probes for Amplification >= 4 AND Minimum Size (Kb) of Region for Amplification >= 0.0 AND Minimum Avg. Absolute Log Ratio for Amplification >= 0.3) OR (Minimum Number of Probes for Deletion >= 4 AND Minimum Size (Kb) of Region for Deletion >= 0.0 AND Minimum Avg. Absolute Log Ratio for Deletion >= 0.3)
Feature Level Filter: gIsSaturated = true OR rIsSaturated = true OR gIsFeatNonUnifOL = true OR rIsFeatNonUnifOL = true OR LogRatio = 0
Design Level Filter: Homology = 0 OR IsPseudoautosomal = 1
Genomic Boundary: OFF
Show Flat Intervals: OFF

AberrationNo	Chr	Cytoband	Start	Stop	#Probes	Amplification	Deletion	pval	Gene Names	omim_feb2016	Hs_hg19_CNV_20120403
C3_ihg2016_252185028566_1_2											
1	chr1	p31.2	69289409	69363038	6	0.000000	-1.011.855	4,09E-24			
2	chr1	p31.1	72768855	72795480	5	0.000000	-3.156.342	2,99E-59			CNV_4229, CNV_0383, CNV_34829, CNV_3293, CNV_34830, CNV_34831, CNV_34832, CNV_34833, CNV_74531, CNV_74532, CNV_84304, CNV_105572, CNV_23231, CNV_97396, CNV_60842, CNV_103487, CNV_22923, CNV_58405, CNV_104750, CNV_111188, CNV_43688, CNV_64030, CNV_6832, CNV_30388, CNV_31627, CNV_1005, CNV_1006, CNV_1543, CNV_37834, CNV_1009, CNV_84305, CNV_74533
3	chr1	q21.3	152556449	152581944	6	0.000000	-1.581.137	8,77E-46	LCE3C	omimGene:612615	CNV_8333, CNV_3314, CNV_53646, CNV_34711, CNV_34712, CNV_0393, CNV_34713, CNV_6786, CNV_74715, CNV_97511, CNV_84576, CNV_107201, CNV_22907, CNV_1558, CNV_103653, CNV_23222, CNV_105006, CNV_112109, CNV_37400, CNV_64960, CNV_38795, CNV_30438, CNV_31661, CNV_38961, CNV_1019, CNV_84577, CNV_74714, CNV_1018, CNV_31662

4	chr2	p11.2 - p11.1	89456779	91766271	39	0.000000	-0.701896	8,96E-79			CNV_34427, CNV_35873, CNV_107994, CNV_115389, CNV_104585, CNV_74122, CNV_59010, CNV_74104, CNV_34620, CNV_115397, CNV_74105, CNV_23246, CNV_4296, CNV_115405, CNV_78647, CNV_89796, CNV_74119, CNV_115399, CNV_7287, CNV_35877, CNV_74116, CNV_89797, CNV_89798, CNV_7288, CNV_89799, CNV_89800, CNV_89801, CNV_74109, CNV_4297, CNV_74103, CNV_104587, CNV_74117, CNV_104588, CNV_115406, CNV_78648, CNV_89802, CNV_89803, CNV_78649, CNV_78650, CNV_89804, CNV_2057, CNV_74126, CNV_74137, CNV_74127, CNV_115408, CNV_74125, CNV_74139, CNV_59011, CNV_104589, CNV_115410, CNV_37972, CNV_104236, CNV_2058, CNV_9954, CNV_50591, CNV_50592, CNV_1591, CNV_74138, CNV_68084, CNV_74128, CNV_78651, CNV_78652, CNV_89805, CNV_89806, CNV_78653, CNV_89807, CNV_89808, CNV_74129, CNV_4298, CNV_9956, CNV_50596, CNV_50597, CNV_50598, CNV_115409, CNV_78656, CNV_1353, CNV_59607, CNV_78654, CNV_74136, CNV_74141, CNV_74130, CNV_59012, CNV_78655, CNV_74131, CNV_74135, CNV_104590, CNV_89809, CNV_89810, CNV_23275, CNV_74140, CNV_68083, CNV_35878, CNV_1355, CNV_1354, CNV_9957, CNV_53731, CNV_35879, CNV_35880, CNV_59013, CNV_74132, CNV_35881, CNV_74133, CNV_30966, CNV_74134, CNV_3375, CNV_74142, CNV_97916, CNV_63132, CNV_22848, CNV_63181, CNV_115411, CNV_32325, CNV_32326, CNV_61793, CNV_23041, CNV_22637, CNV_74143, CNV_30967, CNV_35882, CNV_74147, CNV_74148, CNV_30968, CNV_74149, CNV_35883, CNV_30969, CNV_32327, CNV_35884, CNV_0527, CNV_35885, CNV_44038, CNV_115412, CNV_115413, CNV_115414
5	chr3	q26.1	162514534	162619141	10	0.000000	-3.697.499	5,54E-164			CNV_30185, CNV_4359, CNV_3461, CNV_36095, CNV_0563, CNV_36096, CNV_98511, CNV_43698, CNV_63640, CNV_63641, CNV_104707, CNV_107837, CNV_110712, CNV_79986, CNV_91670, CNV_38805, CNV_103484, CNV_1649, CNV_107609, CNV_31126, CNV_7377, CNV_38965, CNV_32529, CNV_59583, CNV_59584, CNV_59608, CNV_91671, CNV_91672, CNV_110135, CNV_110510, CNV_4360, CNV_110136, CNV_1377, CNV_23278, CNV_59901, CNV_107999, CNV_110714, CNV_4361
6	chr4	q13.2	69387056	69483277	12	0.563394	0.000000	1,85E-15	UGT2B17, UGT2B15	omimMorbid:601903	CNV_3499, CNV_92177, CNV_38138, CNV_104781, CNV_110895, CNV_92178, CNV_106520, CNV_63852, CNV_31177, CNV_7448, CNV_23279, CNV_103525, CNV_38966, CNV_110896, CNV_110897, CNV_92179, CNV_1082, CNV_1083, CNV_1388, CNV_1389, CNV_110898, CNV_1390, CNV_1084, CNV_1085, CNV_1391, CNV_1392, CNV_63853, CNV_106521, CNV_92180, CNV_110900, CNV_110901, CNV_92181, CNV_106522, CNV_1087
7	chr6	p21.32	32455274	32497381	6	1.252.292	0.000000	1,28E-23	HLA-DRB5	omimGene:604776	CNV_3603, CNV_4493, CNV_64476, CNV_7567, CNV_37840, CNV_69469, CNV_69421, CNV_69472, CNV_31280, CNV_4767, CNV_111517, CNV_107632, CNV_111516, CNV_111496, CNV_81335, CNV_81336, CNV_81340, CNV_81342, CNV_81343, CNV_93642, CNV_93646, CNV_103651, CNV_23237, CNV_111505, CNV_111527, CNV_69411, CNV_69414, CNV_32790, CNV_111524, CNV_59326, CNV_37538, CNV_69427, CNV_69442, CNV_69416, CNV_81337, CNV_93647, CNV_69443, CNV_38969, CNV_81344, CNV_111507, CNV_111495, CNV_111529, CNV_69438, CNV_32791, CNV_111531, CNV_69444, CNV_69440, CNV_63176, CNV_69458, CNV_111503, CNV_106274, CNV_103652, CNV_59327, CNV_69445
8	chr7	q33	133589546	133703003	20	0.533676	0.000000	1,22E-23	EXOC4	omimGene:608185	CNV_3704

9	chr7	q34	141766883	141792094	6	0.000000	-0.861591	7,12E-18	MGAM	omimGene:154360	CNV_3706, CNV_64964, CNV_64965, CNV_53811, CNV_31393, CNV_53904, CNV_30265, CNV_9586, CNV_10212, CNV_37566, CNV_52111, CNV_52112, CNV_52113, CNV_53831, CNV_53845, CNV_54002, CNV_52114, CNV_7607, CNV_36626, CNV_52115, CNV_52116, CNV_52117, CNV_112000, CNV_59387, CNV_103758, CNV_32953, CNV_37997, CNV_1744, CNV_10211, CNV_30264, CNV_52118, CNV_52119, CNV_52120, CNV_52121, CNV_52122, CNV_53977, CNV_54016, CNV_52123, CNV_52124, CNV_52125, CNV_52126, CNV_36627, CNV_52127, CNV_31394
10	chr7	q36.2 - q36.3	154682564	155100335	72	0.476541	0.000000	1,01E-69	DPP6, LOC100132707, PAXIP1, LOC202781, HTR5A, INSIG1	omimMorbid:126141, omimGene:601305, omimGene:602055	CNV_0111, CNV_52163, CNV_53472, CNV_32967, CNV_52164, CNV_64996, CNV_95012, CNV_62748, CNV_32968, CNV_2721, CNV_38667, CNV_103763, CNV_64997, CNV_95013, CNV_95015, CNV_31404, CNV_34450, CNV_52165, CNV_100189, CNV_100190, CNV_64998, CNV_64999, CNV_95016, CNV_1173, CNV_52166, CNV_62750, CNV_52167
11	chr8	p11.22	39234992	39386158	28	0.614904	0.000000	2,80E-43	ADAM5P, ADAM3A		CNV_2749, CNV_36815, CNV_105114, CNV_112161, CNV_59473, CNV_36816, CNV_105798, CNV_65114, CNV_106294, CNV_37462, CNV_7683, CNV_82574, CNV_95380, CNV_106595, CNV_31439, CNV_1779, CNV_103790, CNV_106668, CNV_33030, CNV_38096, CNV_1462, CNV_107920, CNV_3728, CNV_1197, CNV_1463, CNV_1198, CNV_112162, CNV_1460, CNV_1199, CNV_1200, CNV_1461, CNV_1465, CNV_95381, CNV_1464, CNV_112166
12	chr14	q11.2	19376762	20420849	35	0.000000	-0.523435	2,92E-40	OR11H12, POTEG, P704P, OR4Q3, OR4M1, OR4N2, OR4K2, OR4K5, OR4K1		CNV_7028, CNV_58655, CNV_76530, CNV_87085, CNV_31953, CNV_71830, CNV_71837, CNV_87086, CNV_87087, CNV_113648, CNV_71851, CNV_71863, CNV_38838, CNV_113649, CNV_71840, CNV_76531, CNV_87088, CNV_37153, CNV_2174, CNV_110092, CNV_71850, CNV_76533, CNV_87091, CNV_87092, CNV_87093, CNV_113650, CNV_106643, CNV_106171, CNV_0812, CNV_2256, CNV_4822, CNV_71852, CNV_4823, CNV_71839, CNV_76534, CNV_76535, CNV_87095, CNV_87096, CNV_76536, CNV_3929, CNV_37621, CNV_71853, CNV_71841, CNV_37154, CNV_106382, CNV_30646, CNV_76537, CNV_76539, CNV_87097, CNV_87098, CNV_71836, CNV_71861, CNV_71833, CNV_71844, CNV_30647, CNV_106383, CNV_76541, CNV_113651, CNV_71854, CNV_76542, CNV_87099, CNV_87102, CNV_87103, CNV_76543, CNV_87104, CNV_87101, CNV_71838, CNV_76544, CNV_76545, CNV_71855, CNV_106384, CNV_87100, CNV_71856, CNV_76546, CNV_87105, CNV_87106, CNV_76547, CNV_76548, CNV_76549, CNV_87107, CNV_71857, CNV_71835, CNV_76550, CNV_87108, CNV_87109, CNV_76552, CNV_87110, CNV_4824, CNV_113652, CNV_71858, CNV_4825, CNV_71842, CNV_76551, CNV_87111, CNV_76553, CNV_76554, CNV_87112, CNV_87114, CNV_87115, CNV_66584, CNV_30648, CNV_87116, CNV_76557, CNV_71832, CNV_106385, CNV_87117, CNV_0316, CNV_71859, CNV_113654, CNV_113655, CNV_38951, CNV_104041, CNV_87118, CNV_3036, CNV_8762, CNV_8763, CNV_8765, CNV_9234, CNV_34547, CNV_34575, CNV_34656, CNV_34660, CNV_34664, CNV_4826, CNV_34659, CNV_8764, CNV_9235, CNV_71831, CNV_71834, CNV_34563, CNV_76556, CNV_71860, CNV_87113, CNV_49301, CNV_49302, CNV_49303, CNV_49304, CNV_76555, CNV_76558, CNV_101886, CNV_101887, CNV_4827, CNV_2175, CNV_66583, CNV_76559, CNV_101888

13	chr14	q32.33	106875948	106931193	12	0.000000	-0.751217	2,01E-26			CNV_10460, CNV_7026, CNV_113813, CNV_35213, CNV_0317, CNV_35219, CNV_35220, CNV_22954, CNV_7027, CNV_1956, CNV_76774, CNV_87415, CNV_72030, CNV_4856, CNV_113829, CNV_72017, CNV_0182, CNV_72049, CNV_72044, CNV_5322, CNV_87418, CNV_4857, CNV_35234, CNV_72010, CNV_102035, CNV_76777, CNV_113836, CNV_72037, CNV_108102, CNV_113862, CNV_72081, CNV_72008, CNV_106381, CNV_87416, CNV_22914, CNV_59724, CNV_23308, CNV_87412, CNV_76778, CNV_113816, CNV_71999, CNV_76779, CNV_76780, CNV_87423, CNV_87424, CNV_87425, CNV_4858, CNV_9735, CNV_9736, CNV_9739, CNV_10455, CNV_10456, CNV_10458, CNV_10459, CNV_49296, CNV_49297, CNV_49298, CNV_53475, CNV_53711, CNV_53925, CNV_53949, CNV_53956, CNV_53963
14	chr15	q11.1 - q11.2	20571586	22173977	56	0.322088	0.000000	3,67E-24	GOLGA6L6, GOLGA8C, BCL8, POTEB, NF1P1, LOC646214, CXADRP2	omimGene:601889	CNV_3951, CNV_3070, CNV_49466, CNV_49467, CNV_49468, CNV_49469, CNV_49470, CNV_49471, CNV_8779, CNV_8780, CNV_8781, CNV_8784, CNV_9251, CNV_34504, CNV_34562, CNV_34576, CNV_9250, CNV_9253, CNV_34608, CNV_34665, CNV_31980, CNV_30670, CNV_76794, CNV_87446, CNV_35284, CNV_0318, CNV_72097, CNV_87450, CNV_105467, CNV_113869, CNV_7046, CNV_34663, CNV_72116, CNV_76802, CNV_37625, CNV_87451, CNV_113870, CNV_0776, CNV_2181, CNV_58702, CNV_72112, CNV_76795, CNV_87443, CNV_35291, CNV_72115, CNV_106392, CNV_113871, CNV_38976, CNV_106645, CNV_76804, CNV_87445, CNV_87453, CNV_35292, CNV_0442, CNV_35293, CNV_72092, CNV_102039, CNV_108468, CNV_23174, CNV_72114, CNV_76805, CNV_76806, CNV_22641, CNV_1957, CNV_39159, CNV_9255, CNV_0443, CNV_0444, CNV_110096, CNV_72111, CNV_4865, CNV_72123, CNV_4866, CNV_72124, CNV_66724, CNV_72142, CNV_76807, CNV_87454, CNV_72143, CNV_72125, CNV_87455, CNV_4867, CNV_87456, CNV_76808, CNV_72126, CNV_72127, CNV_59597, CNV_113872, CNV_34545, CNV_8785, CNV_9254, CNV_72128, CNV_8783, CNV_23136, CNV_113877, CNV_0775, CNV_2180, CNV_4870, CNV_76809, CNV_87457, CNV_87458, CNV_76810, CNV_108479, CNV_9256, CNV_76811, CNV_87459, CNV_87460, CNV_76812, CNV_4871, CNV_76814, CNV_76818, CNV_87462, CNV_87465, CNV_87466, CNV_34607, CNV_72130, CNV_102040, CNV_102041, CNV_72129, CNV_102042, CNV_102043, CNV_9742, CNV_102044, CNV_72121, CNV_113876, CNV_63050, CNV_113874, CNV_113873, CNV_72122, CNV_72141, CNV_113878, CNV_8786, CNV_72120, CNV_72132, CNV_72139, CNV_4863, CNV_72140, CNV_72133, CNV_72134, CNV_87467, CNV_87468, CNV_72135, CNV_72144, CNV_72119, CNV_76815, CNV_72137, CNV_106393, CNV_59725
15	chr15	q22.2	60800311	61056864	60	0.659282	0.000000	3,23E-111	RORA	omimGene:600825	CNV_113955
16	chr20	p13	1563715	1577359	4	0.000000	-2.136.494	5,49E-33	SIRPB1		CNV_4090, CNV_35897, CNV_35898, CNV_0536, CNV_35899, CNV_104442, CNV_114874, CNV_67634, CNV_35900, CNV_104187, CNV_35901, CNV_35902, CNV_35903, CNV_35904, CNV_1522, CNV_7297, CNV_79098, CNV_79099, CNV_90432, CNV_90433, CNV_35905, CNV_110389, CNV_22910, CNV_61903, CNV_107995, CNV_37839, CNV_102897, CNV_90434, CNV_114876, CNV_31029, CNV_32405, CNV_1992, CNV_90435
17	chr21	q22.3	45439340	45623174	42	0.447925	0.000000	2,36E-35	TRAPPC10, PWP2, C21orf33		CNV_7327, CNV_59777, CNV_110451, CNV_103107, CNV_67804, CNV_104492, CNV_43382
18	chrX	p22.33	81548	92387	4	0.000000	-1.150.246	8,39E-22			CNV_73882, CNV_83235, CNV_83236, CNV_96362, CNV_73883, CNV_96363
19	chrX	p21.1	31986625	32863006	188	0.000000	-1.096.913	0.000	DMD	omimMorbid:300377	CNV_52980, CNV_73952, CNV_52981, CNV_1845, CNV_67956
20	chrX	p21.1	32279743	32344451	14	0.000000	-4.563.121	1,14E-139	DMD	omimMorbid:300377	CNV_52980

21	chrY	p11.32	31548	42387	4	0.000000	-1.150.246	8,39E-22			CNV_83894, CNV_97143
C2_ihg2016_252185028566_1_1											
22	chr1	p31.2	69289409	69363038	6	0.000000	-0.969409	1,86E-21			
23	chr1	p31.1	72768855	72795480	5	0.000000	-3.500.261	3,72E-64			CNV_4229, CNV_0383, CNV_34829, CNV_3293, CNV_34830, CNV_34831, CNV_34832, CNV_34833, CNV_74531, CNV_74532, CNV_84304, CNV_105572, CNV_23231, CNV_97396, CNV_60842, CNV_103487, CNV_22923, CNV_58405, CNV_104750, CNV_111188, CNV_43688, CNV_64030, CNV_6832, CNV_30388, CNV_31627, CNV_1005, CNV_1006, CNV_1543, CNV_37834, CNV_1009, CNV_84305, CNV_74533
24	chr1	q21.3	152569857	152581944	5	0.000000	-1.823.880	9,45E-48	LCE3C	omimGene:612615	CNV_8333, CNV_3314, CNV_53646, CNV_34711, CNV_34712, CNV_0393, CNV_34713, CNV_6786, CNV_74715, CNV_97511, CNV_84576, CNV_107201, CNV_22907, CNV_1558, CNV_103653, CNV_23222, CNV_105006, CNV_112109, CNV_37400, CNV_64960, CNV_38795, CNV_30438, CNV_31661, CNV_38961, CNV_84577, CNV_74714, CNV_1018, CNV_31662
25	chr2	p11.2 - p11.1	89456779	91766271	39	0.000000	-0.753662	6,31E-85			CNV_34427, CNV_35873, CNV_107994, CNV_115389, CNV_104585, CNV_74122, CNV_59010, CNV_74104, CNV_34620, CNV_115397, CNV_74105, CNV_23246, CNV_4296, CNV_115405, CNV_78647, CNV_89796, CNV_74119, CNV_115399, CNV_7287, CNV_35877, CNV_74116, CNV_89797, CNV_89798, CNV_7288, CNV_89799, CNV_89800, CNV_89801, CNV_74109, CNV_4297, CNV_74103, CNV_104587, CNV_74117, CNV_104588, CNV_115406, CNV_78648, CNV_89802, CNV_89803, CNV_78649, CNV_78650, CNV_89804, CNV_2057, CNV_74126, CNV_74137, CNV_74127, CNV_115408, CNV_74125, CNV_74139, CNV_59011, CNV_104589, CNV_115410, CNV_37972, CNV_104236, CNV_2058, CNV_9954, CNV_50591, CNV_50592, CNV_1591, CNV_74138, CNV_68084, CNV_74128, CNV_78651, CNV_78652, CNV_89805, CNV_89806, CNV_78653, CNV_89807, CNV_89808, CNV_74129, CNV_4298, CNV_9956, CNV_50596, CNV_50597, CNV_50598, CNV_115409, CNV_78656, CNV_1353, CNV_59607, CNV_78654, CNV_74136, CNV_74141, CNV_74130, CNV_59012, CNV_78655, CNV_74131, CNV_74135, CNV_104590, CNV_89809, CNV_89810, CNV_23275, CNV_74140, CNV_68083, CNV_35878, CNV_1355, CNV_1354, CNV_9957, CNV_53731, CNV_35879, CNV_35880, CNV_59013, CNV_74132, CNV_35881, CNV_74133, CNV_30966, CNV_74134, CNV_3375, CNV_74142, CNV_97916, CNV_63132, CNV_22848, CNV_63181, CNV_115411, CNV_32325, CNV_32326, CNV_61793, CNV_23041, CNV_22637, CNV_74143, CNV_30967, CNV_35882, CNV_74147, CNV_74148, CNV_30968, CNV_74149, CNV_35883, CNV_30969, CNV_32327, CNV_35884, CNV_0527, CNV_35885, CNV_44038, CNV_115412, CNV_115413, CNV_115414
26	chr3	q26.1	162514534	162619141	10	0.000000	-3.802.632	3,73E-168			CNV_30185, CNV_4359, CNV_3461, CNV_36095, CNV_0563, CNV_36096, CNV_98511, CNV_43698, CNV_63640, CNV_63641, CNV_104707, CNV_107837, CNV_110712, CNV_79986, CNV_91670, CNV_38805, CNV_103484, CNV_1649, CNV_107609, CNV_31126, CNV_7377, CNV_38965, CNV_32529, CNV_59583, CNV_59584, CNV_59608, CNV_91671, CNV_91672, CNV_110135, CNV_110510, CNV_4360, CNV_110136, CNV_1377, CNV_23278, CNV_59901, CNV_107999, CNV_110714, CNV_4361
27	chr4	q13.2	69387056	69483277	12	0.582679	0.000000	3,35E-15	UGT2B17, UGT2B15	omimMorbid:601903	CNV_3499, CNV_92177, CNV_38138, CNV_104781, CNV_110895, CNV_92178, CNV_106520, CNV_63852, CNV_31177, CNV_7448, CNV_23279, CNV_103525, CNV_38966, CNV_110896, CNV_110897, CNV_92179, CNV_1082, CNV_1083, CNV_1388, CNV_1389, CNV_110898, CNV_1390, CNV_1084, CNV_1085, CNV_1391, CNV_1392, CNV_63853, CNV_106521, CNV_92180, CNV_110900, CNV_110901, CNV_92181, CNV_106522, CNV_1087

28	chr6	p21.32	32455274	32493043	5	1.428.077	0.000000	2,89E-23	HLA-DRB5	omimGene:604776	CNV_3603, CNV_4493, CNV_64476, CNV_7567, CNV_37840, CNV_69469, CNV_69421, CNV_69472, CNV_31280, CNV_4767, CNV_111517, CNV_107632, CNV_111516, CNV_111496, CNV_81335, CNV_81336, CNV_81340, CNV_81342, CNV_81343, CNV_93642, CNV_93646, CNV_103651, CNV_23237, CNV_111505, CNV_111527, CNV_69411, CNV_69414, CNV_32790, CNV_111524, CNV_59326, CNV_37538, CNV_69427, CNV_69442, CNV_69416, CNV_81337, CNV_93647, CNV_69443, CNV_38969, CNV_81344, CNV_111507, CNV_111495, CNV_111529, CNV_69438, CNV_32791, CNV_111531, CNV_69444, CNV_69440, CNV_63176, CNV_69458, CNV_111503, CNV_106274
29	chr7	q33	133589546	133703003	20	0.567940	0.000000	1,02E-24	EXOC4	omimGene:608185	CNV_3704
30	chr7	q34	141766883	141792094	6	0.000000	-0.943321	3,10E-20	MGAM	omimGene:154360	CNV_3706, CNV_64964, CNV_64965, CNV_53811, CNV_31393, CNV_53904, CNV_30265, CNV_9586, CNV_10212, CNV_37566, CNV_52111, CNV_52112, CNV_52113, CNV_53831, CNV_53845, CNV_54002, CNV_52114, CNV_7607, CNV_36626, CNV_52115, CNV_52116, CNV_52117, CNV_112000, CNV_59387, CNV_103758, CNV_32953, CNV_37997, CNV_1744, CNV_10211, CNV_30264, CNV_52118, CNV_52119, CNV_52120, CNV_52121, CNV_52122, CNV_53977, CNV_54016, CNV_52123, CNV_52124, CNV_52125, CNV_52126, CNV_36627, CNV_52127, CNV_31394
31	chr8	p11.22	39234992	39386158	28	0.645703	0.000000	5,71E-45	ADAM5P, ADAM3A		CNV_2749, CNV_36815, CNV_105114, CNV_112161, CNV_59473, CNV_36816, CNV_105798, CNV_65114, CNV_106294, CNV_37462, CNV_7683, CNV_82574, CNV_95380, CNV_106595, CNV_31439, CNV_1779, CNV_103790, CNV_106668, CNV_33030, CNV_38096, CNV_1462, CNV_107920, CNV_3728, CNV_1197, CNV_1463, CNV_1198, CNV_112162, CNV_1460, CNV_1199, CNV_1200, CNV_1461, CNV_1465, CNV_95381, CNV_1464, CNV_112166
32	chr14	q11.2	19376762	20420849	35	0.000000	-0.566622	9,47E-44	OR11H12, POTEG, P704P, OR4Q3, OR4M1, OR4N2, OR4K2, OR4K5, OR4K1		CNV_7028, CNV_58655, CNV_76530, CNV_87085, CNV_31953, CNV_71830, CNV_71837, CNV_87086, CNV_87087, CNV_113648, CNV_71851, CNV_71863, CNV_38838, CNV_113649, CNV_71840, CNV_76531, CNV_87088, CNV_37153, CNV_2174, CNV_110092, CNV_71850, CNV_76533, CNV_87091, CNV_87092, CNV_87093, CNV_113650, CNV_106643, CNV_106171, CNV_0812, CNV_2256, CNV_4822, CNV_71852, CNV_4823, CNV_71839, CNV_76534, CNV_76535, CNV_87095, CNV_87096, CNV_76536, CNV_3929, CNV_37621, CNV_71853, CNV_71841, CNV_37154, CNV_106382, CNV_30646, CNV_76537, CNV_76539, CNV_87097, CNV_87098, CNV_71836, CNV_71861, CNV_71833, CNV_71844, CNV_30647, CNV_106383, CNV_76541, CNV_113651, CNV_71854, CNV_76542, CNV_87099, CNV_87102, CNV_87103, CNV_76543, CNV_87104, CNV_87101, CNV_71838, CNV_76544, CNV_76545, CNV_71855, CNV_106384, CNV_87100, CNV_71856, CNV_76546, CNV_87105, CNV_87106, CNV_76547, CNV_76548, CNV_76549, CNV_87107, CNV_71857, CNV_71835, CNV_76550, CNV_87108, CNV_87109, CNV_76552, CNV_87110, CNV_4824, CNV_113652, CNV_71858, CNV_4825, CNV_71842, CNV_76551, CNV_87111, CNV_76553, CNV_76554, CNV_87112, CNV_87114, CNV_87115, CNV_66584, CNV_30648, CNV_87116, CNV_76557, CNV_71832, CNV_106385, CNV_87117, CNV_0316, CNV_71859, CNV_113654, CNV_113655, CNV_38951, CNV_104041, CNV_87118, CNV_3036, CNV_8762, CNV_8763, CNV_8765, CNV_9234, CNV_34547, CNV_34575, CNV_34656, CNV_34660, CNV_34664, CNV_4826, CNV_34659, CNV_8764, CNV_9235, CNV_71831, CNV_71834, CNV_34563, CNV_76556, CNV_71860, CNV_87113, CNV_49301, CNV_49302, CNV_49303, CNV_49304, CNV_76555, CNV_76558, CNV_101886, CNV_101887, CNV_4827, CNV_2175, CNV_66583, CNV_76559, CNV_101888

33	chr14	q32.33	106875948	106917229	9	0.000000	-0.925531	1,67E-20			CNV_10460, CNV_7026, CNV_113813, CNV_35213, CNV_0317, CNV_35219, CNV_35220, CNV_22954, CNV_7027, CNV_1956, CNV_76774, CNV_87415, CNV_72030, CNV_4856, CNV_113829, CNV_72017, CNV_0182, CNV_72049, CNV_72044, CNV_5322, CNV_87418, CNV_4857, CNV_35234, CNV_72010, CNV_102035, CNV_76777, CNV_113836, CNV_72037, CNV_108102, CNV_113862, CNV_72081, CNV_72008, CNV_106381, CNV_87416, CNV_22914, CNV_59724, CNV_23308, CNV_87412, CNV_76778, CNV_113816
34	chr15	q22.2	60800311	61066895	62	0.654805	0.000000	9,55E-105	RORA	omimGene:600825	CNV_113955
35	chr20	p13	1563715	1577359	4	0.000000	-1.852.324	5,16E-30	SIRPB1		CNV_4090, CNV_35897, CNV_35898, CNV_0536, CNV_35899, CNV_104442, CNV_114874, CNV_67634, CNV_35900, CNV_104187, CNV_35901, CNV_35902, CNV_35903, CNV_35904, CNV_1522, CNV_7297, CNV_79098, CNV_79099, CNV_90432, CNV_90433, CNV_35905, CNV_110389, CNV_22910, CNV_61903, CNV_107995, CNV_37839, CNV_102897, CNV_90434, CNV_114876, CNV_31029, CNV_32405, CNV_1992, CNV_90435
36	chr21	q22.3	45436248	45623174	43	0.413437	0.000000	2,64E-28	TRAPPC10, PWP2, C21orf33		CNV_7327, CNV_59777, CNV_110451, CNV_103107, CNV_67804, CNV_104492, CNV_43382
37	chrX	p22.33	81548	92387	4	0.000000	-1.153.265	3,40E-20			CNV_73882, CNV_83235, CNV_83236, CNV_96362, CNV_73883, CNV_96363
38	chrX	p21.1	31986625	32863006	188	0.000000	-1.107.216	0.000	DMD	omimMorbid:300377	CNV_52980, CNV_73952, CNV_52981, CNV_1845, CNV_67956
39	chrX	p21.1	32279743	32344451	14	0.000000	-4.606.299	5,67E-145	DMD	omimMorbid:300377	CNV_52980
40	chrY	p11.32	31548	42387	4	0.000000	-1.153.265	3,40E-20			CNV_83894, CNV_97143
41	chrY	p11.31 - p11.2	2700469	9940379	396	0.419644	0.000000	5,64E-92	RPS4Y1, ZFY, TGIF2LY, PCDH11Y, LOC100101121, TTTY23, TSPY2, LOC100101116, TTTY1, TTTY2, LOC100101117, TTTY21, LOC100101115, TTTY7, TTTY7B, TTTY8, TTTY8B, AMELY, TBL1Y, PRKY, TTTY16, TTTY12, TTTY18, TTTY19, TTTY11, RBMY1A3P, TTTY20, FAM197Y2, TSPY4, TSPY3, TSPY1, RBMY3AP, TTTY22	omimGene:470000, omimGene:490000, omimGene:400025, omimGene:400022, omimGene:410000, omimGene:400033, omimGene:400008, omimGene:480100	CNV_31579, CNV_59553, CNV_97163, CNV_4179, CNV_2283, CNV_97165, CNV_2284, CNV_33189, CNV_31580, CNV_83912, CNV_97166, CNV_31581, CNV_31582, CNV_97167, CNV_97169, CNV_37073, CNV_37829, CNV_83913, CNV_97170, CNV_97171, CNV_83914, CNV_97172, CNV_97173, CNV_4180, CNV_83915, CNV_97174, CNV_0829, CNV_2285, CNV_37074, CNV_33191, CNV_83916, CNV_97175, CNV_83917, CNV_31584, CNV_4182, CNV_59885, CNV_97176
C1_ihg2016_252185028565_1_2											
42	chr1	p31.2	69289409	69363038	6	0.000000	-1.003.069	2,33E-24			

43	chr1	p31.1	72768855	72795480	5	0.000000	-3.752.135	3,44E-66			CNV_4229, CNV_0383, CNV_34829, CNV_3293, CNV_34830, CNV_34831, CNV_34832, CNV_34833, CNV_74531, CNV_74532, CNV_84304, CNV_105572, CNV_23231, CNV_97396, CNV_60842, CNV_103487, CNV_22923, CNV_58405, CNV_104750, CNV_111188, CNV_43688, CNV_64030, CNV_6832, CNV_30388, CNV_31627, CNV_1005, CNV_1006, CNV_1543, CNV_37834, CNV_1009, CNV_84305, CNV_74533
44	chr1	q21.3	152569857	152581944	5	0.000000	-1.756.018	6,54E-43	LCE3C	omimGene:612615	CNV_8333, CNV_3314, CNV_53646, CNV_34711, CNV_34712, CNV_0393, CNV_34713, CNV_6786, CNV_74715, CNV_97511, CNV_84576, CNV_107201, CNV_22907, CNV_1558, CNV_103653, CNV_23222, CNV_105006, CNV_112109, CNV_37400, CNV_64960, CNV_38795, CNV_30438, CNV_31661, CNV_38961, CNV_84577, CNV_74714, CNV_1018, CNV_31662
45	chr2	p11.2 - p11.1	89441099	91766271	41	0.000000	-0.650687	2,43E-78			CNV_34427, CNV_35873, CNV_107994, CNV_35875, CNV_115389, CNV_104585, CNV_74122, CNV_59010, CNV_74104, CNV_34620, CNV_115397, CNV_74105, CNV_23246, CNV_4296, CNV_115405, CNV_78647, CNV_89796, CNV_74119, CNV_115399, CNV_7287, CNV_35877, CNV_74116, CNV_89797, CNV_89798, CNV_7288, CNV_89799, CNV_89800, CNV_89801, CNV_74109, CNV_4297, CNV_74103, CNV_104587, CNV_74117, CNV_104588, CNV_115406, CNV_78648, CNV_89802, CNV_89803, CNV_78649, CNV_78650, CNV_89804, CNV_2057, CNV_74126, CNV_74137, CNV_74127, CNV_115408, CNV_74125, CNV_74139, CNV_59011, CNV_104589, CNV_115410, CNV_37972, CNV_104236, CNV_2058, CNV_9954, CNV_50591, CNV_50592, CNV_1591, CNV_74138, CNV_68084, CNV_74128, CNV_78651, CNV_78652, CNV_89805, CNV_89806, CNV_78653, CNV_89807, CNV_89808, CNV_74129, CNV_4298, CNV_9956, CNV_50596, CNV_50597, CNV_50598, CNV_115409, CNV_78656, CNV_1353, CNV_59607, CNV_78654, CNV_74136, CNV_74141, CNV_74130, CNV_59012, CNV_78655, CNV_74131, CNV_74135, CNV_104590, CNV_89809, CNV_89810, CNV_23275, CNV_74140, CNV_68083, CNV_35878, CNV_1355, CNV_1354, CNV_9957, CNV_53731, CNV_35879, CNV_35880, CNV_59013, CNV_74132, CNV_35881, CNV_74133, CNV_30966, CNV_74134, CNV_3375, CNV_74142, CNV_97916, CNV_63132, CNV_22848, CNV_63181, CNV_115411, CNV_32325, CNV_32326, CNV_61793, CNV_23041, CNV_22637, CNV_74143, CNV_30967, CNV_35882, CNV_74147, CNV_74148, CNV_30968, CNV_74149, CNV_35883, CNV_30969, CNV_32327, CNV_35884, CNV_0527, CNV_35885, CNV_44038, CNV_115412, CNV_115413, CNV_115414
46	chr3	q26.1	162514534	162619141	10	0.000000	-3.454.784	1,89E-166			CNV_30185, CNV_4359, CNV_3461, CNV_36095, CNV_0563, CNV_36096, CNV_98511, CNV_43698, CNV_63640, CNV_63641, CNV_104707, CNV_107837, CNV_110712, CNV_79986, CNV_91670, CNV_38805, CNV_103484, CNV_1649, CNV_107609, CNV_31126, CNV_7377, CNV_38965, CNV_32529, CNV_59583, CNV_59584, CNV_59608, CNV_91671, CNV_91672, CNV_110135, CNV_110510, CNV_4360, CNV_110136, CNV_1377, CNV_23278, CNV_59901, CNV_107999, CNV_110714, CNV_4361
47	chr4	q13.2	69387056	69483277	12	0.537361	0.000000	8,57E-16	UGT2B17, UGT2B15	omimMorbid:601903	CNV_3499, CNV_92177, CNV_38138, CNV_104781, CNV_110895, CNV_92178, CNV_106520, CNV_63852, CNV_31177, CNV_7448, CNV_23279, CNV_103525, CNV_38966, CNV_110896, CNV_110897, CNV_92179, CNV_1082, CNV_1083, CNV_1388, CNV_1389, CNV_110898, CNV_1390, CNV_1084, CNV_1085, CNV_1391, CNV_1392, CNV_63853, CNV_106521, CNV_92180, CNV_110900, CNV_110901, CNV_92181, CNV_106522, CNV_1087

48	chr6	p21.32	32455274	32497381	6	1.243.609	0.000000	7,72E-24	HLA-DRB5	omimGene:604776	CNV_3603, CNV_4493, CNV_64476, CNV_7567, CNV_37840, CNV_69469, CNV_69421, CNV_69472, CNV_31280, CNV_4767, CNV_111517, CNV_107632, CNV_111516, CNV_111496, CNV_81335, CNV_81336, CNV_81340, CNV_81342, CNV_81343, CNV_93642, CNV_93646, CNV_103651, CNV_23237, CNV_111505, CNV_111527, CNV_69411, CNV_69414, CNV_32790, CNV_111524, CNV_59326, CNV_37538, CNV_69427, CNV_69442, CNV_69416, CNV_81337, CNV_93647, CNV_69443, CNV_38969, CNV_81344, CNV_111507, CNV_111495, CNV_111529, CNV_69438, CNV_32791, CNV_111531, CNV_69444, CNV_69440, CNV_63176, CNV_69458, CNV_111503, CNV_106274, CNV_103652, CNV_59327, CNV_69445
49	chr7	q33	133594273	133695190	18	0.571454	0.000000	7,54E-28	EXOC4	omimGene:608185	CNV_3704
50	chr7	q34	141771233	141792094	5	0.000000	-0.989797	4,54E-20	MGAM	omimGene:154360	CNV_3706, CNV_64964, CNV_64965, CNV_53811, CNV_31393, CNV_53904, CNV_30265, CNV_9586, CNV_10212, CNV_37566, CNV_52111, CNV_52112, CNV_52113, CNV_53831, CNV_53845, CNV_54002, CNV_52114, CNV_7607, CNV_52115, CNV_52116, CNV_52117, CNV_112000, CNV_59387, CNV_103758, CNV_32953, CNV_37997, CNV_1744, CNV_10211, CNV_30264, CNV_52118, CNV_52119, CNV_52120, CNV_52121, CNV_52122, CNV_53977, CNV_54016, CNV_52123, CNV_52124, CNV_52125, CNV_52126, CNV_36627, CNV_52127, CNV_31394
51	chr8	p11.22	39234992	39386158	28	0.609156	0.000000	9,55E-48	ADAM5P, ADAM3A		CNV_2749, CNV_36815, CNV_105114, CNV_112161, CNV_59473, CNV_36816, CNV_105798, CNV_65114, CNV_106294, CNV_37462, CNV_7683, CNV_82574, CNV_95380, CNV_106595, CNV_31439, CNV_1779, CNV_103790, CNV_106668, CNV_33030, CNV_38096, CNV_1462, CNV_107920, CNV_3728, CNV_1197, CNV_1463, CNV_1198, CNV_112162, CNV_1460, CNV_1199, CNV_1200, CNV_1461, CNV_1465, CNV_95381, CNV_1464, CNV_112166

52	chr14	q11.2	19376762	20420849	35	0.000000	-0.509071	1,97E-42	OR11H12, POTEG, P704P, OR4Q3, OR4M1, OR4N2, OR4K2, OR4K5, OR4K1	CNV_7028, CNV_58655, CNV_76530, CNV_87085, CNV_31953, CNV_71830, CNV_71837, CNV_87086, CNV_87087, CNV_113648, CNV_71851, CNV_71863, CNV_38838, CNV_113649, CNV_71840, CNV_76531, CNV_87088, CNV_37153, CNV_2174, CNV_110092, CNV_71850, CNV_76533, CNV_87091, CNV_87092, CNV_87093, CNV_113650, CNV_106643, CNV_106171, CNV_0812, CNV_2256, CNV_4822, CNV_71852, CNV_4823, CNV_71839, CNV_76534, CNV_76535, CNV_87095, CNV_87096, CNV_76536, CNV_3929, CNV_37621, CNV_71853, CNV_71841, CNV_37154, CNV_106382, CNV_30646, CNV_76537, CNV_76539, CNV_87097, CNV_87098, CNV_71836, CNV_71861, CNV_71833, CNV_71844, CNV_30647, CNV_106383, CNV_76541, CNV_113651, CNV_71854, CNV_76542, CNV_87099, CNV_87102, CNV_87103, CNV_76543, CNV_87104, CNV_87101, CNV_71838, CNV_76544, CNV_76545, CNV_71855, CNV_106384, CNV_87100, CNV_71856, CNV_76546, CNV_87105, CNV_87106, CNV_76547, CNV_76548, CNV_76549, CNV_87107, CNV_71857, CNV_71835, CNV_76550, CNV_87108, CNV_87109, CNV_76552, CNV_87110, CNV_4824, CNV_113652, CNV_71858, CNV_4825, CNV_71842, CNV_76551, CNV_87111, CNV_76553, CNV_76554, CNV_87112, CNV_87114, CNV_87115, CNV_66584, CNV_30648, CNV_87116, CNV_76557, CNV_71832, CNV_106385, CNV_87117, CNV_0316, CNV_71859, CNV_113654, CNV_113655, CNV_38951, CNV_104041, CNV_87118, CNV_3036, CNV_8762, CNV_8763, CNV_8765, CNV_9234, CNV_34547, CNV_34575, CNV_34656, CNV_34660, CNV_34664, CNV_4826, CNV_34659, CNV_8764, CNV_9235, CNV_71831, CNV_71834, CNV_34563, CNV_76556, CNV_71860, CNV_87113, CNV_49301, CNV_49302, CNV_49303, CNV_49304, CNV_76555, CNV_76558, CNV_101886, CNV_101887, CNV_4827, CNV_2175, CNV_66583, CNV_76559, CNV_101888
53	chr14	q32.33	106875948	106931193	12	0.000000	-0.728304	3,25E-27		CNV_10460, CNV_7026, CNV_113813, CNV_35213, CNV_0317, CNV_35219, CNV_35220, CNV_22954, CNV_7027, CNV_1956, CNV_76774, CNV_87415, CNV_72030, CNV_4856, CNV_113829, CNV_72017, CNV_0182, CNV_72049, CNV_72044, CNV_5322, CNV_87418, CNV_4857, CNV_35234, CNV_72010, CNV_102035, CNV_76777, CNV_113836, CNV_72037, CNV_108102, CNV_113862, CNV_72081, CNV_72008, CNV_106381, CNV_87416, CNV_22914, CNV_59724, CNV_23308, CNV_87412, CNV_76778, CNV_113816, CNV_71999, CNV_76779, CNV_76780, CNV_87423, CNV_87424, CNV_87425, CNV_4858, CNV_9735, CNV_9736, CNV_9739, CNV_10455, CNV_10456, CNV_10458, CNV_10459, CNV_49296, CNV_49297, CNV_49298, CNV_53475, CNV_53711, CNV_53925, CNV_53949, CNV_53956, CNV_53963

54	chr15	q11.1 - q11.2	20571586	22173977	56	0.312967	0.000000	5,08E-27	GOLGA6L6, GOLGA8C, BCL8, POTEB, NF1P1, LOC646214, CXADRP2	omimGene:601889	CNV_3951, CNV_3070, CNV_49466, CNV_49467, CNV_49468, CNV_49469, CNV_49470, CNV_49471, CNV_8779, CNV_8780, CNV_8781, CNV_8784, CNV_9251, CNV_34504, CNV_34562, CNV_34576, CNV_9250, CNV_9253, CNV_34608, CNV_34665, CNV_31980, CNV_30670, CNV_76794, CNV_87446, CNV_35284, CNV_0318, CNV_72097, CNV_87450, CNV_105467, CNV_113869, CNV_7046, CNV_34663, CNV_72116, CNV_76802, CNV_37625, CNV_87451, CNV_113870, CNV_0776, CNV_2181, CNV_58702, CNV_72112, CNV_76795, CNV_87443, CNV_35291, CNV_72115, CNV_106392, CNV_113871, CNV_38976, CNV_106645, CNV_76804, CNV_87445, CNV_87453, CNV_35292, CNV_0442, CNV_35293, CNV_72092, CNV_102039, CNV_108468, CNV_23174, CNV_72114, CNV_76805, CNV_76806, CNV_22641, CNV_1957, CNV_39159, CNV_9255, CNV_0443, CNV_0444, CNV_110096, CNV_72111, CNV_4865, CNV_72123, CNV_4866, CNV_72124, CNV_66724, CNV_72142, CNV_76807, CNV_87454, CNV_72143, CNV_72125, CNV_87455, CNV_4867, CNV_87456, CNV_76808, CNV_72126, CNV_72127, CNV_59597, CNV_113872, CNV_34545, CNV_8785, CNV_9254, CNV_72128, CNV_8783, CNV_23136, CNV_113877, CNV_0775, CNV_2180, CNV_4870, CNV_76809, CNV_87457, CNV_87458, CNV_76810, CNV_108479, CNV_9256, CNV_76811, CNV_87459, CNV_87460, CNV_76812, CNV_4871, CNV_76814, CNV_76818, CNV_87462, CNV_87465, CNV_87466, CNV_34607, CNV_72130, CNV_102040, CNV_102041, CNV_72129, CNV_102042, CNV_102043, CNV_9742, CNV_102044, CNV_72121, CNV_113876, CNV_63050, CNV_113874, CNV_113873, CNV_72122, CNV_72141, CNV_113878, CNV_8786, CNV_72120, CNV_72132, CNV_72139, CNV_4863, CNV_72140, CNV_72133, CNV_72134, CNV_87467, CNV_87468, CNV_72135, CNV_72144, CNV_72119, CNV_76815, CNV_72137, CNV_106393, CNV_59725
55	chr15	q22.2	60800311	61056864	60	0.636268	0.000000	6,24E-115	RORA	omimGene:600825	CNV_113955
56	chr20	p13	1563715	1577359	4	0.000000	-1.976.575	6,40E-35	SIRPB1		CNV_4090, CNV_35897, CNV_35898, CNV_0536, CNV_35899, CNV_104442, CNV_114874, CNV_67634, CNV_35900, CNV_104187, CNV_35901, CNV_35902, CNV_35903, CNV_35904, CNV_1522, CNV_7297, CNV_79098, CNV_79099, CNV_90432, CNV_90433, CNV_35905, CNV_110389, CNV_22910, CNV_61903, CNV_107995, CNV_37839, CNV_102897, CNV_90434, CNV_114876, CNV_31029, CNV_32405, CNV_1992, CNV_90435
57	chr21	q22.3	45445332	45617046	40	0.458105	0.000000	2,41E-39	TRAPPC10, PWP2, C21orf33		CNV_7327, CNV_59777
58	chrX	p22.33	81548	92387	4	0.000000	-1.145.587	8,49E-22			CNV_73882, CNV_83235, CNV_83236, CNV_96362, CNV_73883, CNV_96363
59	chrX	p21.1	31986625	32863006	188	0.000000	-1.087.190	0.000	DMD	omimMorbid:300377	CNV_52980, CNV_73952, CNV_52981, CNV_1845, CNV_67956
60	chrX	p21.1	32279743	32344451	14	0.000000	-4.561.102	1,05E-144	DMD	omimMorbid:300377	CNV_52980
61	chrY	p11.32	31548	42387	4	0.000000	-1.145.587	8,49E-22			CNV_83894, CNV_97143
wt_ihg2016_252185028565_1_1											
62	chr1	p31.1	72768855	72795480	5	0.000000	-3.582.584	7,29E-61			CNV_4229, CNV_0383, CNV_34829, CNV_3293, CNV_34830, CNV_34831, CNV_34832, CNV_34833, CNV_74531, CNV_74532, CNV_84304, CNV_105572, CNV_23231, CNV_97396, CNV_60842, CNV_103487, CNV_22923, CNV_58405, CNV_104750, CNV_111188, CNV_43688, CNV_64030, CNV_6832, CNV_30388, CNV_31627, CNV_1005, CNV_1006, CNV_1543, CNV_37834, CNV_1009, CNV_84305, CNV_74533

63	chr1	q21.3	152556449	152581944	6	0.000000	-1.914.994	2,15E-59	LCE3C	omimGene:612615	CNV_8333, CNV_3314, CNV_53646, CNV_34711, CNV_34712, CNV_0393, CNV_34713, CNV_6786, CNV_74715, CNV_97511, CNV_84576, CNV_107201, CNV_22907, CNV_1558, CNV_103653, CNV_23222, CNV_105006, CNV_112109, CNV_37400, CNV_64960, CNV_38795, CNV_30438, CNV_31661, CNV_38961, CNV_1019, CNV_84577, CNV_74714, CNV_1018, CNV_31662
64	chr2	p11.2 - p11.1	89450514	91766271	40	0.000000	-0.629424	3,43E-72			CNV_34427, CNV_35873, CNV_107994, CNV_115389, CNV_104585, CNV_74122, CNV_59010, CNV_74104, CNV_34620, CNV_115397, CNV_74105, CNV_23246, CNV_4296, CNV_115405, CNV_78647, CNV_89796, CNV_74119, CNV_115399, CNV_7287, CNV_35877, CNV_74116, CNV_89797, CNV_89798, CNV_7288, CNV_89799, CNV_89800, CNV_89801, CNV_74109, CNV_4297, CNV_74103, CNV_104587, CNV_74117, CNV_104588, CNV_115406, CNV_78648, CNV_89802, CNV_89803, CNV_78649, CNV_78650, CNV_89804, CNV_2057, CNV_74126, CNV_74137, CNV_74127, CNV_115408, CNV_74125, CNV_74139, CNV_59011, CNV_104589, CNV_115410, CNV_37972, CNV_104236, CNV_2058, CNV_9954, CNV_50591, CNV_50592, CNV_1591, CNV_74138, CNV_68084, CNV_74128, CNV_78651, CNV_78652, CNV_89805, CNV_89806, CNV_78653, CNV_89807, CNV_89808, CNV_74129, CNV_4298, CNV_9956, CNV_50596, CNV_50597, CNV_50598, CNV_115409, CNV_78656, CNV_1353, CNV_59607, CNV_78654, CNV_74136, CNV_74141, CNV_74130, CNV_59012, CNV_78655, CNV_74131, CNV_74135, CNV_104590, CNV_89809, CNV_89810, CNV_23275, CNV_74140, CNV_68083, CNV_35878, CNV_1355, CNV_1354, CNV_9957, CNV_53731, CNV_35879, CNV_35880, CNV_59013, CNV_74132, CNV_35881, CNV_74133, CNV_30966, CNV_74134, CNV_3375, CNV_74142, CNV_97916, CNV_63132, CNV_22848, CNV_63181, CNV_115411, CNV_32325, CNV_32326, CNV_61793, CNV_23041, CNV_22637, CNV_74143, CNV_30967, CNV_35882, CNV_74147, CNV_74148, CNV_30968, CNV_74149, CNV_35883, CNV_30969, CNV_32327, CNV_35884, CNV_0527, CNV_35885, CNV_44038, CNV_115412, CNV_115413, CNV_115414
65	chr3	q26.1	162514534	162619141	10	0.000000	-3.739.900	4,33E-215			CNV_30185, CNV_4359, CNV_3461, CNV_36095, CNV_0563, CNV_36096, CNV_98511, CNV_43698, CNV_63640, CNV_63641, CNV_104707, CNV_107837, CNV_110712, CNV_79986, CNV_91670, CNV_38805, CNV_103484, CNV_1649, CNV_107609, CNV_31126, CNV_7377, CNV_38965, CNV_32529, CNV_59583, CNV_59584, CNV_59608, CNV_91671, CNV_91672, CNV_110135, CNV_110510, CNV_4360, CNV_110136, CNV_1377, CNV_23278, CNV_59901, CNV_107999, CNV_110714, CNV_4361
66	chr4	q13.2	69387056	69483277	12	0.571615	0.000000	5,26E-18	UGT2B17, UGT2B15	omimMorbid:601903	CNV_3499, CNV_92177, CNV_38138, CNV_104781, CNV_110895, CNV_92178, CNV_106520, CNV_63852, CNV_31177, CNV_7448, CNV_23279, CNV_103525, CNV_38966, CNV_110896, CNV_110897, CNV_92179, CNV_1082, CNV_1083, CNV_1388, CNV_1389, CNV_110898, CNV_1390, CNV_1084, CNV_1085, CNV_1391, CNV_1392, CNV_63853, CNV_106521, CNV_92180, CNV_110900, CNV_110901, CNV_92181, CNV_106522, CNV_1087

67	chr6	p21.32	32455274	32497381	6	1.058.279	0.000000	5,42E-19	HLA-DRB5	omimGene:604776	CNV_3603, CNV_4493, CNV_64476, CNV_7567, CNV_37840, CNV_69469, CNV_69421, CNV_69472, CNV_31280, CNV_4767, CNV_111517, CNV_107632, CNV_111516, CNV_111496, CNV_81335, CNV_81336, CNV_81340, CNV_81342, CNV_81343, CNV_93642, CNV_93646, CNV_103651, CNV_23237, CNV_111505, CNV_111527, CNV_69411, CNV_69414, CNV_32790, CNV_111524, CNV_59326, CNV_37538, CNV_69427, CNV_69442, CNV_69416, CNV_81337, CNV_93647, CNV_69443, CNV_38969, CNV_81344, CNV_111507, CNV_111495, CNV_111529, CNV_69438, CNV_32791, CNV_111531, CNV_69444, CNV_69440, CNV_63176, CNV_69458, CNV_111503, CNV_106274, CNV_103652, CNV_59327, CNV_69445
68	chr7	q33	133589546	133695190	19	0.546312	0.000000	4,63E-27	EXOC4	omimGene:608185	CNV_3704
69	chr7	q34	141766883	141792094	6	0.000000	-0.823517	1,83E-17	MGAM	omimGene:154360	CNV_3706, CNV_64964, CNV_64965, CNV_53811, CNV_31393, CNV_53904, CNV_30265, CNV_9586, CNV_10212, CNV_37566, CNV_52111, CNV_52112, CNV_52113, CNV_53831, CNV_53845, CNV_54002, CNV_52114, CNV_7607, CNV_36626, CNV_52115, CNV_52116, CNV_52117, CNV_112000, CNV_59387, CNV_103758, CNV_32953, CNV_37997, CNV_1744, CNV_10211, CNV_30264, CNV_52118, CNV_52119, CNV_52120, CNV_52121, CNV_52122, CNV_53977, CNV_54016, CNV_52123, CNV_52124, CNV_52125, CNV_52126, CNV_36627, CNV_52127, CNV_31394
70	chr8	p11.22	39234992	39386158	28	0.643860	0.000000	1,17E-53	ADAM5P, ADAM3A		CNV_2749, CNV_36815, CNV_105114, CNV_112161, CNV_59473, CNV_36816, CNV_105798, CNV_65114, CNV_106294, CNV_37462, CNV_7683, CNV_82574, CNV_95380, CNV_106595, CNV_31439, CNV_1779, CNV_103790, CNV_106668, CNV_33030, CNV_38096, CNV_1462, CNV_107920, CNV_3728, CNV_1197, CNV_1463, CNV_1198, CNV_112162, CNV_1460, CNV_1199, CNV_1200, CNV_1461, CNV_1465, CNV_95381, CNV_1464, CNV_112166
71	chr14	q11.2	19376762	20420849	35	0.000000	-0.525227	2,24E-45	OR11H12, POTEG, P704P, OR4Q3, OR4M1, OR4N2, OR4K2, OR4K5, OR4K1		CNV_7028, CNV_58655, CNV_76530, CNV_87085, CNV_31953, CNV_71830, CNV_71837, CNV_87086, CNV_87087, CNV_113648, CNV_71851, CNV_71863, CNV_38838, CNV_113649, CNV_71840, CNV_76531, CNV_87088, CNV_37153, CNV_2174, CNV_110092, CNV_71850, CNV_76533, CNV_87091, CNV_87092, CNV_87093, CNV_113650, CNV_106643, CNV_106171, CNV_0812, CNV_2256, CNV_4822, CNV_71852, CNV_4823, CNV_71839, CNV_76534, CNV_76535, CNV_87095, CNV_87096, CNV_76536, CNV_3929, CNV_37621, CNV_71853, CNV_71841, CNV_37154, CNV_106382, CNV_30646, CNV_76537, CNV_76539, CNV_87097, CNV_87098, CNV_71836, CNV_71861, CNV_71833, CNV_71844, CNV_30647, CNV_106383, CNV_76541, CNV_113651, CNV_71854, CNV_76542, CNV_87099, CNV_87102, CNV_87103, CNV_76543, CNV_87104, CNV_87101, CNV_71838, CNV_76544, CNV_76545, CNV_71855, CNV_106384, CNV_87100, CNV_71856, CNV_76546, CNV_87105, CNV_87106, CNV_76547, CNV_76548, CNV_76549, CNV_87107, CNV_71857, CNV_71835, CNV_76550, CNV_87108, CNV_87109, CNV_76552, CNV_87110, CNV_4824, CNV_113652, CNV_71858, CNV_4825, CNV_71842, CNV_76551, CNV_87111, CNV_76553, CNV_76554, CNV_87112, CNV_87114, CNV_87115, CNV_66584, CNV_30648, CNV_87116, CNV_76557, CNV_71832, CNV_106385, CNV_87117, CNV_0316, CNV_71859, CNV_113654, CNV_113655, CNV_38951, CNV_104041, CNV_87118, CNV_3036, CNV_8762, CNV_8763, CNV_8765, CNV_9234, CNV_34547, CNV_34575, CNV_34656, CNV_34660, CNV_34664, CNV_4826, CNV_34659, CNV_8764, CNV_9235, CNV_71831, CNV_71834, CNV_34563, CNV_76556, CNV_71860, CNV_87113, CNV_49301, CNV_49302, CNV_49303, CNV_49304, CNV_76555, CNV_76558, CNV_101886, CNV_101887, CNV_4827, CNV_2175, CNV_66583, CNV_76559, CNV_101888

72	chr14	q24.3	74001651	74022324	4	0.000000	-1.157.905	5,82E-20	HEATR4, ACOT1	omimGene:614313	CNV_3942, CNV_35265, CNV_87268, CNV_35266, CNV_105452, CNV_113740, CNV_35267, CNV_66664, CNV_35268, CNV_30658, CNV_76673, CNV_76674, CNV_87266, CNV_87267, CNV_87269, CNV_108324, CNV_35269, CNV_58686, CNV_101980, CNV_71916, CNV_104060, CNV_37822, CNV_106175, CNV_113737, CNV_113739, CNV_113741, CNV_76672, CNV_38974, CNV_7039, CNV_66665, CNV_71917, CNV_31974, CNV_22908, CNV_71918, CNV_23304, CNV_113743
73	chr15	q22.2	60800311	61056864	60	0.649368	0.000000	4,55E-120	RORA	omimGene:600825	CNV_113955
74	chr20	p13	1563715	1577359	4	0.000000	-2.317.592	4,92E-37	SIRPB1		CNV_4090, CNV_35897, CNV_35898, CNV_0536, CNV_35899, CNV_104442, CNV_114874, CNV_67634, CNV_35900, CNV_104187, CNV_35901, CNV_35902, CNV_35903, CNV_35904, CNV_1522, CNV_7297, CNV_79098, CNV_79099, CNV_90432, CNV_90433, CNV_35905, CNV_110389, CNV_22910, CNV_61903, CNV_107995, CNV_37839, CNV_102897, CNV_90434, CNV_114876, CNV_31029, CNV_32405, CNV_1992, CNV_90435
75	chr21	q22.3	45445332	45623174	41	0.509400	0.000000	7,54E-50	TRAPPC10, PWP2, C21orf33		CNV_7327, CNV_59777, CNV_110451, CNV_103107, CNV_67804, CNV_104492, CNV_43382
76	chrX	p22.33	81548	92387	4	0.000000	-0.997456	4,71E-17			CNV_73882, CNV_83235, CNV_83236, CNV_96362, CNV_73883, CNV_96363
77	chrX	p21.1	31986625	32863006	188	0.000000	-1.098.318	0.000	DMD	omimMorbid:300377	CNV_52980, CNV_73952, CNV_52981, CNV_1845, CNV_67956
78	chrX	p21.1	32279743	32344451	14	0.000000	-4.758.613	3,73E-151	DMD	omimMorbid:300377	CNV_52980
79	chrY	p11.32	31548	42387	4	0.000000	-0.997456	4,71E-17			CNV_83894, CNV_97143

Number of calls per sample:
C3_ihg2016_252185028566_1_2 =21
C2_ihg2016_252185028566_1_1 =20
C1_ihg2016_252185028565_1_2 =20
wt_ihg2016_252185028565_1_1 =18

Supplementary Figure S8: Array-CGH of gene corrected hPi-derived cell lines

A

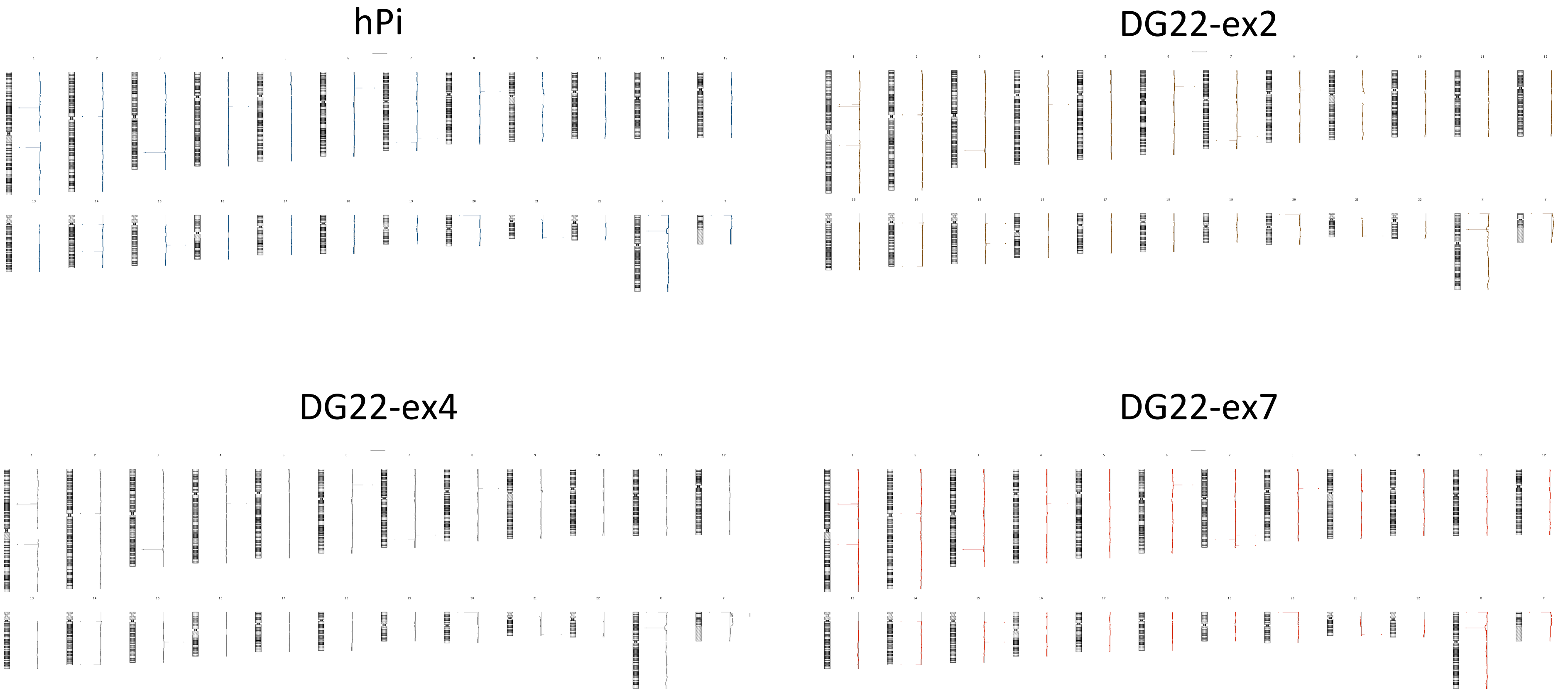

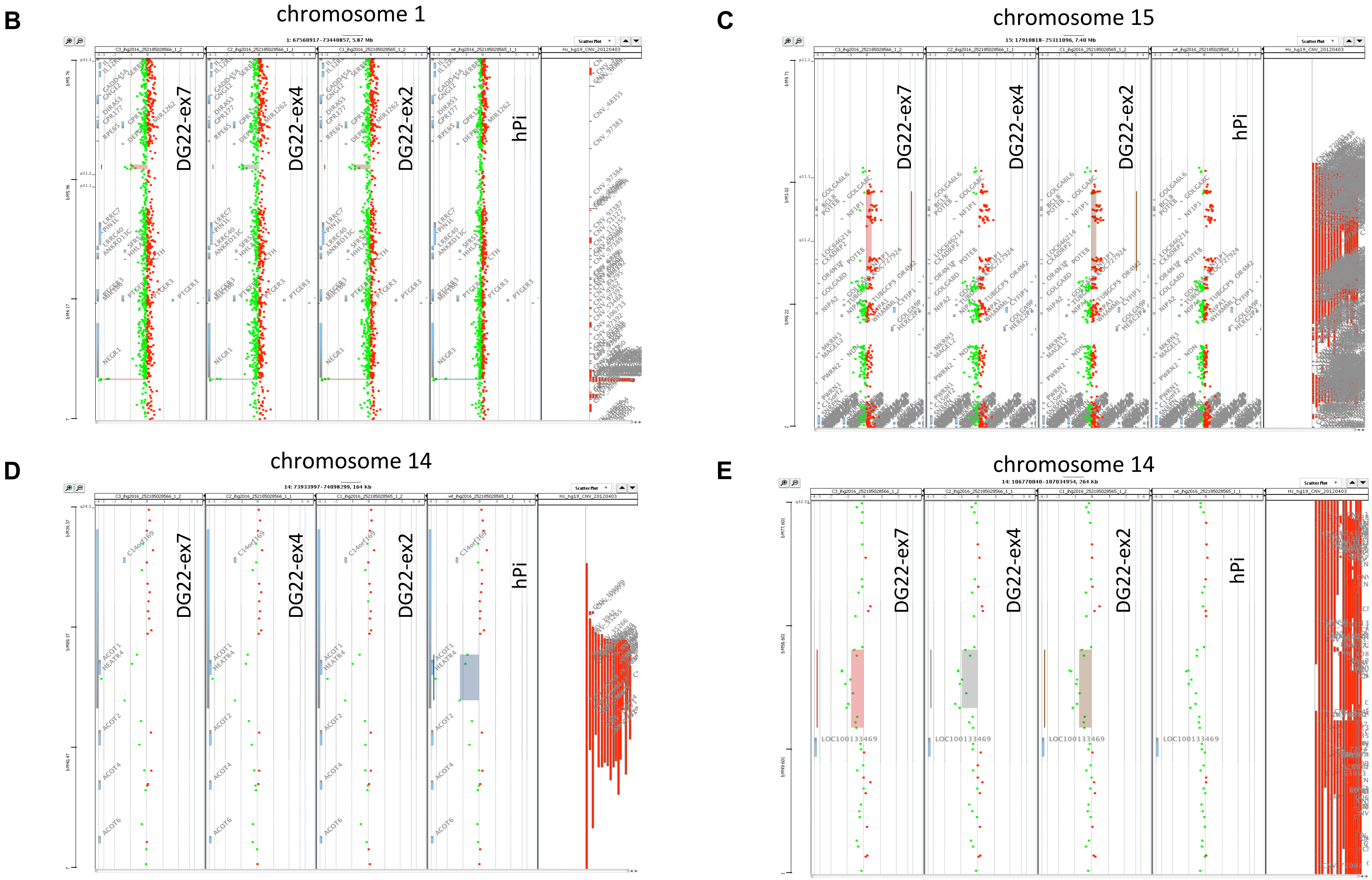

Supplementary Figure S8: Array-CGH of gene corrected hPi-derived cell lines (A) Overview on over all chromosomes. **(B)** Visible difference between hPi and gene corrected cells on non-coding region of chromosome 1. **(C-E)** Differences on chromosomes 14 and 15 detected by applied threshold settings with no visual difference.

Supplementary Experimental Procedures:

Cloning and plasmids

The promoter-less plasmid tGIP, encoding for a truncated version of eGFP followed by an IRES-puro cassette was purchased from Addgene (Addgene Plasmid #26864) and was used in all reporter assays as a donor, as previously described [1]. For cloning of the lentiviral CAG.eGFP-IRES-puro (Lenti CGIP) vector, tGIP was linearized by a partial Acc65I digest for 120 s followed by BsrGI digest. The resulting 1320 bp fragment encompassing the IRES-puro cassette was then ligated into the BsrGI-digested lentiviral vector pRRL.PPT.CAG.GFP.pre2 [2] and resulting clones were analyzed for correct orientation by AgeI + BsrGI double-digest. For cloning of the lentiviral PGK.eGFP-IRES-puro (Lenti PGIP) vector, the full PGK-EM7 cassette was amplified from the pMCS-AAT_PB-PGKpuroTK plasmid (Wellcome Trust Sanger, [3]) using 'PGK ClaI for' and 'PGK XhoI rev' adapter-primers and inserted into pRRL.PPT.CAG.GFP.pre2 by a XhoI + ClaI digest, replacing the CAG promoter and resulting in 'Lenti PGK-EM7-GFP'. A fragment encompassing IRES-puro-PRE-SIN was generated by sequential SfiI and BsrGI digest of Lenti CGIP and ligated into Lenti PGK-EM7-GFP digested with the same enzymes resulting in Lenti PGIP.

For cloning of lentiviral vectors harboring the Z-AAT or M-AAT target sequences inside of the interrupted eGFP cassette (Lenti CiG-ZAAT-t-IP and Lenti CiG-MAAT-t-IP) the whole fragments encompassing the first 327 bp of eGFP followed by a TAA stop codon and the 59 bp target sequences for Z-AAT or M-AAT (**Supplementary Table S4**), followed by the remaining 393 bp of eGFP were synthesized by Eurofins Genomics (Ebersberg, Germany). Both sequences were flanked by AgeI restriction site at the 5' end and SgrDI site at the 3' end and cloned in place of the eGFP in the Lenti CGIP vector. TTR target sequence was cloned from a gene synthesis where TTR target sequence **(Supplementary Table S4)** was added after the TAA stop codon. Synthesized sequence included the AvaI cloning site present in Lenti CiG-ZAAT-t-IP before the interrupted GFP (iGFP), as well as a part of eGFP, and an AscI site present at the end of the ZAAT target sequence and was cloned using AvaI + AscI to replace the Z-AAT target sequence in Lenti CiG-ZAAT-t-IP, resulting in Lenti CiG-TTR-t-IP reporter vector. SOD1 target sequence was ordered as gene synthesis as well, including an AgeI site which was also present at the beginning of the TTR target sequence in Lenti CiG-TTR-t-IP and an AscI site at the end of the target sequence **(Supplementary Table S4)**. AgeI and AscI digest was then used to replace TTR target sequence in Lenti CiG-TTR-t-IP, resulting in Lenti-CiG-SOD1-t-IP reporter vector.

Plasmids encoding for hCas9 and hCas9_D10A, hCas9m2 and hCas9m4 were purchased from Addgene (Addgene Plasmids #41815, #41816, #47314, #47316, [4,5]). Plasmid encoding for hCas9_H840A was generated by BamHI + BstXI digest and ligation of plasmids hCas9 (backbone) and hCas9m2 (insert). Plasmid encoding for hCas9_N863A was generated by BamHI + BstXI digest of hCas9 (backbone), BamHI + BaeGI digest of hCas9 (insert #1) and BaeGI + BstXI digest of hCas9m4 (insert #2) followed by a triple ligation in a 1:3:3 molar ratio. Sequences encoding for the U6-promoter driven AAT_g1, AAT_g2, AAT_g3 and AAT_g4 gRNAs followed by the sgRNA scaffold were synthesized by Eurofins Genomics, Ebersberg, Germany and inserted into the pEX-A2 vector plasmid (Eurofins Genomics). Sequences of U6 promoter and sgRNA scaffold were adapted from Mali *et al.*, 2013 [4] and G+19 bp guide sequences inserted in between U6 promoter and sgRNA scaffold are given in **Supplementary Table S4**. TTR_g1-g4 and SOD1_g1-g5 gRNA expression vectors were cloned in a similar manner. Plasmid pCMV-hyPBase for overexpression of hyperactive piggyBac transposase was obtained from Wellcome Trust Sanger [3].

For cloning of the donor plasmids AAT-PB-CG2Ap*Δtk* and AAT-PB-CD2Ap*Δtk*, we first cloned the joints of the eGFP and DsRedEx fluorescence markers to the puromycin resistance over a T2A-self-cleaving peptidase site. Therefore, we first performed a PCR amplifying the T2A site from a previously published polycistronic lentiviral reprogramming vector, RRL.PPT.SF.hOct34.hKlf4.hSox2.i2dTomato.pre [2], using ‘T2A BsrGI for’ and ‘T2A Tth111I rev’ primers. The latter is a mega-primer which joins the beginning of the puromycin-resistance cassette to the end of the T2A site. The PCR amplicon was digested by BsrGI + Tth111I and ligated into lenti CGIP vector digested with the same enzymes, thereby replacing the IRES cassette with a T2A site, resulting in ‘Lenti CG2AP’. For joining of the DsRedEx to puromycin via T2A site, we first deleted the SexAI site inside the DsRedEx coding sequence by silent mutation using PCR with primers ‘DsRed AgeI for’ and ‘DsRed SexAI mut rev’. The amplicon was digested using AgeI + MscI and inserted into a lentiviral plasmid encoding a SFFV promoter-driven DsRedEx sequence (pRRL.PPT.SF.DsRedEx.pre.snabi.H1AAT3, a kind gift from Prof. Axel Schambach, Hannover Medical School, Hannover, Germany), resulting in ‘Lenti SFFV-DsRed_SexAI_mut’. The cloned DsRedEx sequence with a mutated SexAI site was then amplified again with ‘DsRed AgeI for’ and ‘DsRed + YK rev’ primers, thereby removing the stop codon and adding a BsrGI site including the codons for amino acids YK to DsRedEx, in order to leave the joint to the T2A sequence unchanged when comparing to the GFP-T2A-puro cassette. This amplicon was then digested using AgeI + BsrGI and ligated into Lenti CG2AP digested by the same enzymes, thereby replacing the eGFP cassette by DsRedEx, resulting in ‘Lenti CD2AP’. Using fluorescence microscopy and FACS analysis, both

plasmids (Lenti CG2AP and Lenti CD2AP) were successfully tested for proper expression of fluorescence markers upon transfection of HEK 293T cells. Also both plasmids conveyed resistance to 3 µg/ml puromycin in transiently transfected HEK 293T cells. As a next step we have first exchanged the PGK promoter by the CAG promoter in the pMCS-AAT_PB-PGKpuroTK plasmid. Therefore, we amplified the CMV and chicken beta actin – rabbit globin intron (CBA) elements of the CAG promoter separately using the plasmid pCAGGS2 eGFP as template (a kind gift from Dr. Robert Zweigerdt, MHH, Hannover, Germany). Amplification of the full CAG sequence was not working due to the high CG-content of the CAG promoter. For CMV i/e we used primers ‘CMV NheI for’ and ‘CMV MluI rev’, for the CBA element we used primers ‘CBA AscI for‘ and ‘CBA PciI rev’ Both amplicons were ligated into the TOPO vector pCR2.1 (Invitrogen) and analyzed for orientation of the insertion. TOPO-CMV i/e was then digested using BamHI + MluI, whereas TOPO-CBA was digested using BamHI + AscI. The ~BamHI-CMV i/e-MluI~ fragment was then inserted into the TOPO-CBA, thereby adding the CMV i/e element and deleting the AscI restriction site, resulting in ‘TOPO-CAG(M/A)’. Next, we deleted the BsiWI site in the puromycin coding region of pMCS-AAT_PB-PGKpuroTK by silent mutation using PCR amplification by primers ‘PGK AgeI for’ and ‘puro change RsrII rev’. The amplicon was digested using AgeI + RsrII enzymes and ligated into pMCS-AAT_PB-PGKpuroTK digested by the same enzymes, resulting in pMCS-AAT_PB-PGKpuro_mut_BsiWI_TK. At this point it is important to mention that in parallel to the work presented here, we were also preparing constructs for targeting of the human TTR locus for another project. The route for the fewest total cloning steps for both projects led over an intermediate construct in which the left flanking arm was from the AAT sequence, whereas the right arm was from the TTR sequence. Therefore, we digested a plasmid containing the sequence of the right TTR flanking arm using BsiWI + PacI and ligated it into AAT_PB-PGKpuro_mut_BsiWI_TK digested by the same enzymes, thereby replacing the right AAT flanking arm by the right TTR flanking arm, resulting in the intermediate construct termed ‘AAT left - TTR right’. The CAG sequence was then excised from ‘TOPO-CAG(M/A)’ using NheI + PciI enzymes and ligated into ‘AAT left – TTR right’ digested by NheI + NcoI, thereby replacing the PGK promoter by the CAG promoter, resulting in ‘AAT left–CAG–TTR right’. The right AAT flanking arm was then switched back into the construct by digesting pMCS-AAT_PB-PGKpuroTK and ‘AAT left–CAG–TTR right’ with PacI and NotI, resulting in ‘pMCS-AAT-PB-CAGpuroTK’. Next, Lenti CG2AP vector was digested using NdeI and RsrII enzymes and ligated into ‘pMCS-AAT-PB-CAGpuroTK’ digested by the same enzymes, thereby adding the eGFP-T2A sequence in front of the puro*Δtk* cassette, resulting in AAT-PB-CG2Ap*Δtk*. Finally, we digested ‘Lenti CD2AP’ using AgeI + SexAI and ligated it into AAT-PB-CG2Ap*Δtk*, thereby replacing the eGFP cassette by DsRedEx, resulting in AAT-PB-CD2Ap*Δtk*.

All PCRs for cloning and analysis of genomic sequences except for amplification of the GC-rich CBA element the were performed using Phusion Hot Start II DNA Polymerase with HF-buffer (Thermo Scientific #F549L) according to manufacturer's procedures. The CBA element was amplified by touch-down PCR using Taq-polymerase with 10x $(NH_4)_2SO_4$ buffer (Fermentas, Thermo Scientific), 1 µM $MgCl_2$, 0.2 nM dNTPs, 0.2 µM each primer and 10 % DMSO. Touchdown PCR was started by preheating to 95°C (hot start) and 10 min initial denaturation, followed by 22 cycles of 95°C denaturation for 30s, 65°C (-0.35°C/cycle) touchdown annealing for 30s and 72°C extension for 130s and then by 10 cycles of 95°C for 30s, 55°C for 30s and 72°C for 130s. Cloning primer sequences can be found in **Supplementary Table S4**. All cloned PCR products as well as southern blot probes were analyzed and confirmed by sequencing.

RNA isolation, cDNA synthesis and TaqMan® qRT PCR

RNA from differentiated or pluripotent cells was isolated using the peqGOLD Total RNA Kit (Peqlab) and subsequently used for cDNA synthesis using the SuperScript™ First-Strand Synthesis System (Invitrogen), both according to manufacturer's instructions. CDNA was used for qRT-PCR analysis with TaqMan® probes from Applied Biosystems on the StepOne Plus cycler and all values were normalized to GAPDH. The following probes were used for the analyzed genes:

GAPDH: Hs02758991_g1; AFP: Hs01040595_g1; ALB: Hs00609403_m1; CK18: Hs01941416_g1; CYP1A1: Hs01054797_g1; SERPINA1 (AAT): Hs01097800_m1; TTR: Hs00174914_m1; OCT4: Hs03005111_g1; SOX2: Hs01053049_s1; NANOG: Hs00609403_m1
